# Multi-omics and experimental evidence in human chondrocytes identify caspase-8 as a non-apoptotic regulator of inflammatory, senescent, and fibrotic signaling in osteoarthritis

**DOI:** 10.1186/s12964-026-02985-y

**Published:** 2026-06-06

**Authors:** Jian Mei, Penghui Wei, Nicole Schäfer, Marianne Ehrnsperger, Brian Johnstone, Eva Matalova, Susanne Grässel

**Affiliations:** 1 Department of Orthopedic Surgery, Experimental Orthopedics, Center for Medical Biotechnology (ZMB), University of Regensburg, ZMB im Biopark 1, Am Biopark 9, Regensburg, Germany; 2 Department of Neurosurgery, Neurosurgery Research Institute, The First Affiliated Hospital of Fujian Medical University, Fuzhou, 350005 China; 3 Department of Neurosurgery, National Regional Medical Center, Binhai Campus of the First Affiliated Hospital, Fujian Medical University, Fuzhou, 350212 China; 4 Department of Trauma Surgery and Sporthopaedicum, University of Regensburg, Regensburg, Germany; 5 Department of Orthopedics and Rehabilitation, Oregon Health & Science University, Portland, OR USA; 6 Institute of Animal Physiology and Genetics, Czech Academy of Sciences, Brno, Czech Republic; 7 Department of Physiology, University of Veterinary Sciences Brno, Brno, Czech Republic

**Keywords:** Osteoarthritis, Caspase-8, Chondrocytes, Cellular senescence, Inflammatory signaling, Fibrotic remodeling, Multi-Omics

## Abstract

**Background:**

Osteoarthritis (OA) is characterized by chronic inflammation, cellular senescence, and progressive cartilage remodeling, yet effective disease-modifying therapeutic targets remain limited. Caspase-8 has classically been regarded as a key initiator of the extrinsic apoptotic pathway; however, accumulating evidence suggests that it exerts broad non-apoptotic regulatory functions in a highly context- and cellular state–dependent manner. Here, we systematically investigated the role of caspase-8 in OA pathogenesis and its therapeutic potential.

**Methods:**

We integrated bulk RNAseq, single-cell RNAseq, and spatial transcriptomics databases, in vitro genetic knockdown, inhibitor titration, caspase activity assays and functional assays, quantitative proteomics, in silico protein–protein docking analysis and population-level causal inference using two-sample Mendelian randomization (MR) and SMR/HEIDI analyses to construct and validate a caspase-8–centered regulatory network in human OA chondrocytes.

**Results:**

Across two independent transcriptomic cohorts (GSE168505 and GSE246425), the Death-Inducing Signaling Complex (DISC)–caspase-8 axis and a caspase-8 activation signature were significantly upregulated in osteoarthritic cartilage and in senescent (late passage) OA chondrocytes, and were strongly associated with inflammatory and senescence-related programs. Additional analyses of aging, murine destabilization of the medial meniscus (DMM), and spatial transcriptomic datasets (GSE287861, GSE26475, and GSE254844) highlighted context-dependent CASP8 patterns, including senescence- but not inflammation-associated CASP8 elevation in naturally aged cartilage, no consistent CASP8 expression or activation-score increase after DMM surgery, and regionally heterogeneous CASP8 distribution across cartilage zones. Single-cell RNA sequencing (GSE255460) localized these signatures to OA-expanded inflammatory and fibrocartilage-like chondrocyte subpopulations, in which high CASP8 gene expression defined a distinct transcriptional state characterized by suppression of hyaline cartilage matrix and metabolic programs, together with enrichment of TNFα/interferon and TGF-β–associated inflammatory–fibrotic signaling. CASP8-high chondrocytes also displayed altered inferred ligand–receptor communication, particularly involving extracellular matrix-, adhesion-, and growth factor-related interactions. Stratification by caspase-8 activation potential recapitulated these transcriptional features and revealed induction of senescence-associated secretory phenotype (SASP) programs across multiple chondrocyte lineages. Functionally, pharmacological inhibition of caspase-8 with Z-IETD-FMK improved metabolic activity, reduced cellular senescence and MMP-13 secretion without inducing apoptosis, and partially restored proliferation and migration in OA chondrocytes under inflammatory conditions, with less effects in non-OA cells and no effects in siRNA mediated CASP8 knocked down cells. Quantitative proteomics demonstrated that caspase-8 inhibition attenuated inflammatory, senescence, and canonical NF-κB signaling without suppressing apoptosis, while reshaping proteostasis–chromatin regulatory networks and dampening fibrocartilage-like matrix remodeling. In silico protein–protein docking further showed favorable predicted binding affinities between caspase-8 and selected proteomically altered candidates, such as TGF-β3 and MMP13. Finally, MR and SMR/HEIDI analyses supported CASP8 as a causal risk regulator for knee OA, influenced by multi-tissue expression and epigenetic regulation.

**Conclusions:**

Collectively, our findings identify caspase-8 as a central non-apoptotic signaling hub that couples inflammatory–senescence circuits with fibrotic remodeling in chondrocytes. Consequently, caspase-8 represents a promising genetic and pharmacological therapeutic target, warranting further drug development and translational investigation.

**Supplementary Information:**

The online version contains supplementary material available at 10.1186/s12964-026-02985-y.

## Introduction

Osteoarthritis (OA) is a common chronic degenerative joint disease characterized by chondrocyte dysfunction, extracellular matrix (ECM) remodeling, and persistent low-grade inflammation [1]. Despite its high prevalence, current pharmacological therapies remain largely symptomatic, underscoring the urgent need for disease-modifying treatments [2, 3]. OA can be etiologically categorized into primary and secondary forms [4]. Primary OA is predominantly attributed to age-related degenerative changes and genetic predisposition [5], whereas secondary OA arises from identifiable precipitating factors such as traumatic joint injury [6]. Regardless, its progression ultimately converges on chronic inflammatory activation and chondrocyte signaling disturbances.

Given the central involvement of chondrocyte stress and cell death in OA progression, apoptotic regulators have long been considered potential therapeutic targets [7]. In this context, caspase-8 has previously been proposed as a candidate target based on its canonical role in mediating apoptosis [8]. In an ACLT rabbit OA model, broad-spectrum caspase inhibition reduces chondrocyte apoptosis and attenuates cartilage degeneration, supporting the concept that limiting chondrocyte apoptosis may confer cartilage protection. However, selective inhibition of caspase-8 alone shows limited efficacy, whereas combined inhibition of caspase-8 and caspase-3 reduce the severity of cartilage lesions [9]. These findings raise important mechanistic questions regarding the role of caspase-8 in OA.

In line with a context-dependent complexity, current understanding of caspase-8 functions has expanded to encompass both canonical and non-canonical roles. At the canonical level, caspase-8 is classically activated through death receptor–proximal signaling. Upon engagement of receptors such as TNFR1 or FAS, adaptor proteins including TRADD and FADD are recruited to assemble receptor-associated signaling complexes, most notably the death-inducing signaling complex (DISC) [10, 11]. DISC is responsible for transducing death ligand stimulation of the death receptors CD95, TRAIL-R1 or TRAIL-R2 to apoptosis. This process represents the classical involvement of caspase-8 as a core component of the DISC.

In recent years, increasing attention has been directed toward the non-canonical functions of caspase-8. Accumulating evidence indicates that caspase-8 functions extend far beyond apoptosis, encompassing regulation of inflammatory signaling, immune responses, and cellular differentiation [12]. Importantly, these non-canonical functions can be uncoupled from caspase-8 catalytic activity and are shaped by alternative splicing and post-translational modifications, highlighting the context-dependent and multifunctional nature of caspase-8 signaling [12–14]. This paradigm is well established in immune cells, where caspase-8 predominantly supports non-canonical regulatory programs - such as inflammatory and stress-adaptive signaling - rather than apoptosis [12, 15].

However, in human OA, it remains unclear whether caspase-8 primarily contributes to apoptotic regulation or instead exerts disease-relevant effects predominantly through apoptosis-independent, non-canonical, and context-dependent regulatory functions, such as inflammatory signaling, stress-adaptive responses, and tissue remodeling, or a combination of both. Equally unknown is whether caspase-8 represents a causal driver of OA or merely a secondary responder during disease progression. This lack of mechanistic and genetic clarity fundamentally limits the evaluation of caspase-8 as a therapeutic target. In this context, an integrative multi-layered framework offers a promising entry point for addressing this gap.

Based on these considerations, we hypothesize that the role of caspase-8 in OA pathogenesis undergoes a functional shift beyond its canonical regulation of apoptosis. Under chronic inflammatory conditions characteristic of OA, the apoptosis-independent, non-canonical functions of caspase-8 may predominate in chondrocytes and play a more central pathogenic role. These functions may be regulated at multiple levels, including transcriptional regulation, activation state, and epigenetic or post-translational mechanisms such as alternative splicing and DNA methylation.

To test this hypothesis, we integrated multi-omics analyses, genetic causal inference, and in vitro functional experiments with the following objectives: (1) to characterize the expression and activation patterns of caspase-8 and its regulatory DISC axis in chondrocytes; (2) to evaluate the genetic causal association of caspase-8 signaling with OA susceptibility; and (3) to investigate the impact of caspase-8 activity on chondrocyte functions—including cell viability, apoptosis, proliferation, wound closure, and senescence—as well as its broader regulatory effects on molecular signaling networks, thereby dissecting the relative contributions of its canonical and non-canonical functions in healthy- and OA- chondrocytes.

## Materials and methods

### Study design

**Fig. 1:**
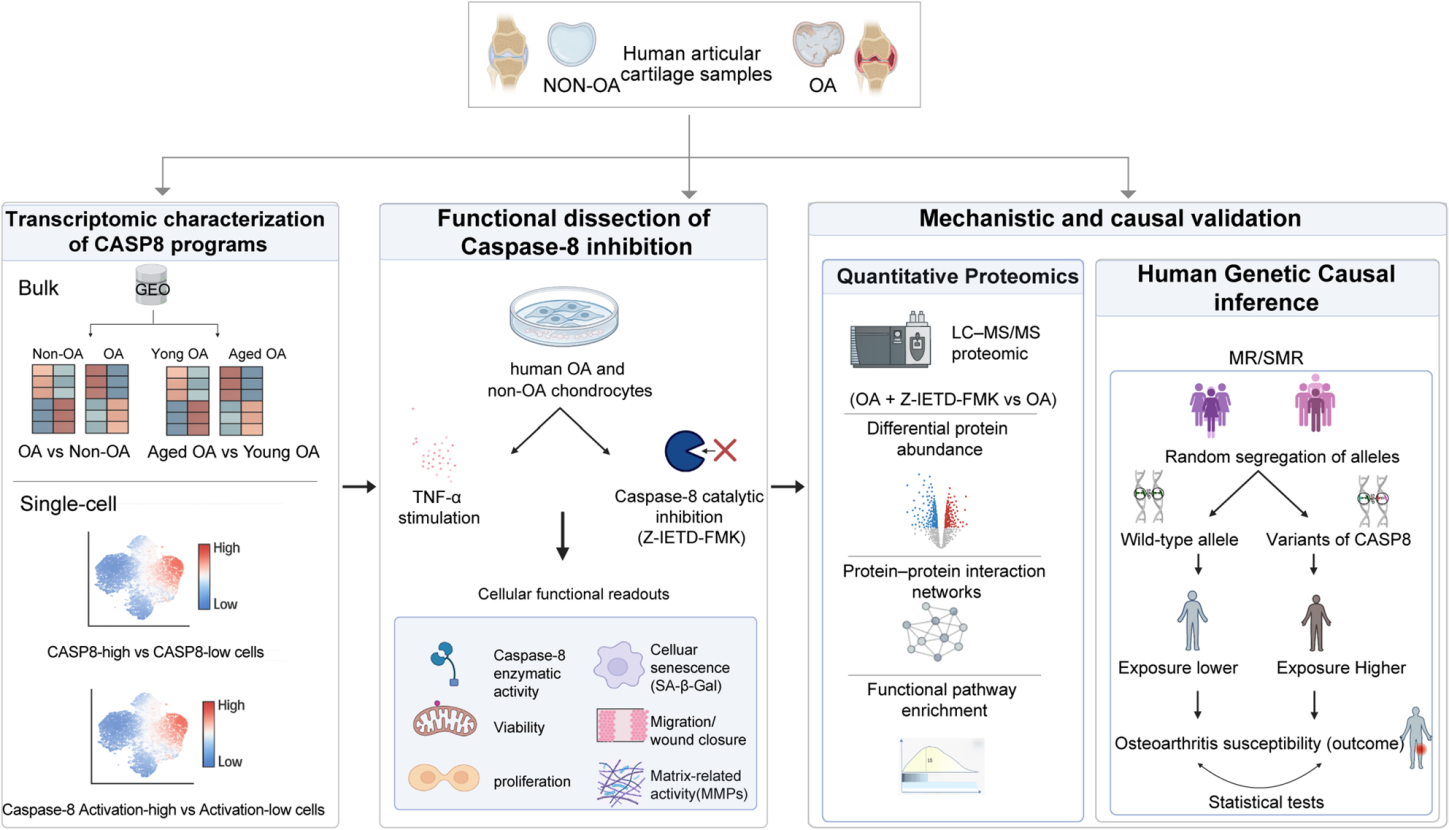
Study Framework and Multi-omics Integration Strategy. Schematic overview of the systems approach used to investigate caspase-8 in osteoarthritis. The study integrates (1) transcriptomic profiling of public human cartilage datasets (bulk and single-cell RNA-seq), (2) functional validation in primary human OA- and non-OA chondrocytes using the caspase-8 specific inhibitor Z-IETD-FMK (assessing viability, proliferation, senescence, migration, and matrix remodeling), (3) high-resolution quantitative proteomics to map downstream signaling networks, and (4) causal inference using two-sample Mendelian Randomization (MR) and Summary-data-based MR (SMR) to link CASP8 genetics to OA susceptibility

Figure 1 illustrates the study framework. We applied a triangulation approach to investigate the role of caspase-8 in OA and its therapeutic potential. First, transcriptomic analyses of public datasets characterized CASP8 gene expression and related regulators in OA versus non-OA human chondrocytes. Second, functional assays in an in vitro OA model evaluated the effects of caspase-8 activity inhibition (Z-IETD-FMK) on cell metabolism, including viability, proliferation, senescence, wound closure, and matrix-degrading enzyme (MMP) production. Proteomics further elucidated downstream signaling networks. Third, Mendelian randomization analyses leveraging eQTLs, mQTLs, sQTLs, and genetic predictors of circulating caspase-8 protein levels across multiple tissues, including chondrocytes, were conducted to evaluate the causal association between CASP8 gene expression and OA risk. Additional public aging, experimental OA, and spatial transcriptomic datasets were incorporated to contextualize CASP8-related patterns across biological settings, while ligand–receptor communication analysis, in silico CASP8 perturbation, and protein–protein docking were used as complementary computational analyses.

### Sample collection and ethical approval

Cartilage samples were collected from two groups: OA patients undergoing total knee replacement surgery (Asklepiosklinikum, Bad Abbach, Germany) and healthy individuals through cadaveric donations (OHSU, Portland, USA) using leftover sections of clinically certified, pristine cartilage from osteochondral allografts. The OA patients had an average age of 66.4 ± 7.2 years (ranging from 56 to 79 years), with 73.2% being female. In comparison, non-OA cartilage was obtained from five cadavers, with an average age of 24 ± 6.3 years (ranging from 17 to 34 years) and 20.1% female donors. The use of human OA material has been approved by the local ethics committee (No. 22-2915-101; Ethikkommission, University of Regensburg, email: ethikkommission@ur.de), and the written consent of all OA patients has been obtained.

### Isolation and culture of chondrocytes

Chondrocytes were isolated as published previously [16]. The cartilage tissue from all donors was separated from the subchondral bone and cut into small pieces (~ 1–2 mm³). The pieces were digested in type II collagenase (Worthington, Lakewood, NJ, USA) (11 mg collagenase per 1 g cartilage) dissolved in Dulbecco’s Modified Eagle Medium (DMEM, #D8437, Sigma, UK) at 37 °C for 16 h. After digestion, the cell suspension was centrifuged at 300xg for 5 min. The non-OA chondrocytes were then frozen and shipped to Germany on dry ice. All chondrocytes were seeded into T175 culture flasks at a density of 5,000–10,000 cells/cm² and cultured in DMEM F12 medium (Sigma Aldrich, Taufkirchen) supplemented with 10% fetal calf serum (FCS) (Sigma Aldrich, Taufkirchen) and 1% penicillin-streptomycin (Sigma Aldrich, Taufkirchen), with the medium replaced every 2–3 days. Once the cells reached 80–90% confluency, they were passaged. Passage 2–3 of all chondrocytes were used for the experiments.

### In Vitro OA low-grade inflammation model and caspase-8 inhibition intervention

#### Establishment of the TNF-α-based low-grade inflammation model

Human articular chondrocytes (both OA and non-OA) were seeded in 6-well plates at 1 × 10^4^ cells per well and treated with TNF-α (1 or 5 ng/mL) (Life Technologies, Darmstadt) for 24 h. Untreated cells served as controls. After treatment, cells and supernatants were collected for subsequent analyses. At the experimental endpoint, caspase-8 expression was assessed at both the mRNA and protein levels. Protein expression levels of caspase-8 were quantified using enzyme-linked immunosorbent assay (ELISA) kits, with analyses performed on cell lysates (see 3.6). Experiments using 3D pellet cultures were additionally performed as a supplementary comparison, as described in the Supplementary Methods and Supplementary Figure.1.

#### Caspase-8 inhibition

Chondrocytes were treated with Z-IETD-FMK (100 µM) (Bio-Techne, Wiesbaden-Nordenstadt) for 72 h in total as published previously [17]. A 10 mM DMSO stock (Carl Roth, Karlsruhe) was diluted in culture medium to the working concentration. Cells were pre-treated for 48 h with the inhibitor, then co-treated with 1 ng/mL TNF-α for further 24 h to evaluate the functional effects of caspase-8 inhibition.

In addition, we analyzed the effects of different incubation times (12 h, 24 h, 48 h and 72 h) and concentrations (10µM, 25µM, 50µM and 100µM) of the caspase-8 inhibitor Z-IETD-FMK on caspase-8 activity. For that, OA chondrocytes (1 × 10⁴ cells per well) were seeded in opaque white 96-well plates (Thermo Scientific, Nunc™, Cat. #136101) and caspase-8 enzymatic activity was assessed by using caspase-8-Glo luminescent assays (details see 3.4.3).

#### Assessment of caspase-1, -3/7,-8 and − 9 enzymatic activity

Caspases activity was evaluated using the Caspase-Glo^®^ luminescent assays (Promega, Walldorf, caspase-1: Cat. G9952; caspase-3/7: Cat. G8091; caspase-8: Cat. #G8201 and caspase-9: Cat, G8211). Chondrocytes (1 × 10⁴ cells per well) were seeded in opaque white 96-well plates (Thermo Scientific, Nunc™, Cat. #136101) and cultured in 100 µL of DMEM F12 medium plus 10% FCS plus 5% Pen-Strep (Sigma Aldrich, Taufkirchen). After completing the indicated treatments, an equal volume (100 µL) of the Caspase-Glo^®^ reagents was added directly to the culture wells The plates were gently agitated and incubated for 1 h at room temperature to allow signal development. Luminescence was detected using a microplate luminometer (Molecular Devices, Munich), and relative luminescence units (RLUs) were used as a quantitative indicator of caspases activity. Untreated cells (without TNF-α or Z-IETD-FMK) served as baseline controls, and blank wells were included to correct for background signal.

#### Apo-ONE caspase-3/7 assay

The Apo-One Homogeneous fluorigenic Caspase-3/7 Assay (Promega, Cat. G7791) was applied according to manufacturer’s protocol for analysis of caspase-3/7 activity correlating to apoptotic activity in cells, Briefly, 8 × 10^4^ chondrocytes were treated with TNF-α (1ng/mL or 5ng/mL) for 4 h, 12 h and 24 h and cultured in 100 µl DMEM F12 + 10% FCS + 5% Pen-Strep per 96-wells (Thermo Scientific, Nunc™, Cat. #136101). Subsequently, the cell suspension was diluted with 100 µl Apo-ONE reagent. Fluorescence was measured with 360 nm excitation and 465 nm emission using ELISA Reader SpectraMax iD3 (Molecular Devices, Sunnyvale, CA, USA) in duplicate.

#### Annexin V/7-ADD FACS analysis

We performed cellular staining using an Annexin V-FITC antibody (Invitrogen, Cat. A13199) and 7-AAD nucleic acid dye (BD Biosciences, Cat. 559925) for analysis of cell apoptosis by flow cytometry. Chondrocytes kept in 25 cm^2^ flasks, pre-treated with 1 or 5 ng/mL TNFα for 12–24 h, were washed carefully with ice-cold PBS and were subsequently detached with 2mL accutase (Merck, Cat. A6964) on ice and after adding DMEM F12 medium plus 10% FCS, centrifuged for 5 min at 300xg. The cells were counted, then the cell density was adjusted to ∼1 × 10^6^ cells/mL with the Annexin-binding buffer. 100 µL (= 1 × 10^5^ cells) of the solution was incubated with either 5 µL Annexin V-FITC antibody or 5 µL 7-AAD dye or both substances together for 15 min in the dark on ice. Finally, 400 µL Annexin Binding Buffer was carefully mixed with the cell solution on ice and directly analyzed by FACS (Attune NxT, Invitrogen).

### PCR detection

#### Primers

Gene-specific primer pairs were designed using NCBI Primer-BLAST and synthesized by Microsynth, Switzerland (HPLC purification grade). Primer sequences with corresponding references are summarized in Table 1.

**Table 1:** Primer sequences for target and reference genes

Gene	NIH official gene name	Forward Primer (5’-3’)	Reverse Primer (5’-3’)
CASP8	Caspase 8	AGATGGCCACTGTGAACTATG	CCTGTTCTGTGAAGCCTTGT
HPRT1	Hypoxanthine phosphoribosyltransferase 1	TGACACTGGCAAAACAATGCA	GGTCCTTTTCACCAGCAAGCT
TFRC	Transferrin receptor	CAGTCACCGTGTCTTCCTCA	GCTGCCTTCAAACTCATCCG

#### RNA Extraction and qRT-PCR analysis

Total RNA was extracted from chondrocytes using the RNeasy Mini Kit (Qiagen, Cat. No. 74104). RNA quality (A260/280 = 1.8–2.0) and concentration were measured with a Thermo Scientific spectrophotometer (Thermo Scientific, Darmstadt, Germany). cDNA was generated from equal RNA amounts using the AffinityScript QPCR cDNA Synthesis Kit (Agilent Technologies). qRT-PCR was performed with Brilliant III Ultra-Fast SYBR reagents on the MX3005P system (Agilent Technologies). Cycling was run at 95 °C for 2 min, followed by 40 cycles of 95 °C for 5 s and 60 °C for 10 s. Relative gene expression was calculated by the ΔΔCt method and normalized to HPRT1 and TFRC; all samples were analyzed in duplicate.

#### siRNA transfection

Human chondrocytes were seeded in 6 well-plates with a density of 5 × 10^4^ cells per well and grown for two days. According to the manufacturer`s protocol (Dharmacon/Horizon, USA, Lafayette, CO), cells were cultured in antibiotic-free DMEM F12 medium for 24 h. Consequently, 25 nM of the siRNA and non-target (nt)-RNA prepared as described by the manufacturer, was added to the cells for 24 h (ON-TARGETplus non-targeting Pool, #D-001810-10-05, ON-TARGETplus human CASP8 (841) siRNA-SMARTpool, #L-003466-00-0005 and DharmaFECT 1 Transfection Reagent, #T-2001-01). The next day fresh DMEM F12 medium was added to the cells and after 24 h cells were trypsinized and seeded for the functional assays in the absence or presence of 24 h TNF-α stimulation.

### ELISA detection

Caspase-8 protein levels were quantified using the Human Caspase-8 ELISA Kit (Invitrogen, ThermoFisher Scientific, Cat. No. BMS2024). All procedures followed the manufacturer’s instructions. Samples were assayed in duplicate, and absorbance was measured at 450 nm using a microplate reader (Molecular Devices, Munich, Germany). Concentrations were calculated from standard curves generated using a four-parameter logistic (4-PL) model.

### Functional analyses and MMP quantification

Experimental conditions and methods were adapted from a previously published study [16]. In short, cell viability, proliferation, senescence, apoptosis, gap closure (cell migration), and MMP secretion were evaluated under basal conditions (no TNF-α) or TNF-α stimulation (1 ng/mL), in the presence or absence of caspase-8 inhibitor (Z-IETD-FMK, 100 µM) or after siRNA mediated knockdown of caspase-8 (without migration). Cell viability was measured using the CellTiter-Blue^®^ assay (Promega, #G8081) after 72 h of treatment, following the manufacturer’s protocol. Fluorescence was read at 570/600 nm using a microplate reader (Molecular Devices, Munich, Germany). Proliferation was quantified with a BrdU ELISA kit (Roche, #11647229001) with a 10 µM labeling step during the final 24 h. Absorbance was detected at 450 nm. Senescence was assessed using the SA-β-Gal Activity Assay (Cell Biolabs, #CBA231). Fluorescence was quantified at Ex/Em 360/465 nm. Apoptosis was assessed using the Apo-ONE Homogeneous Caspase-3/7 assay (Promega, Fitchburg, WI, USA) according to the manufacturer’s instructions. Migration was analyzed using ibidi culture inserts (ibidi, #80206). Scratch closure was imaged over 0–96 h and quantified with ImageJ (NIH). MMPs (MMP-1/2/3/13, etc.) were measured in culture supernatants using the PROCARTAPLEX^®^ 6-plex Human MMP Panel (ThermoFisher Scientific) on a Bio-Plex 200 (Bio-Rad), analyzed using 5-PL regression. All measurements were conducted in duplicate or triplicate as specified by the manufacturer.

### Public transcriptomic and single-cell analyses

#### Bulk RNA-seq analysis

Bulk RNA-seq datasets of human articular cartilage were obtained from the Gene Expression Omnibus (GEO), including GSE168505 (OA- vs. non-OA cartilage) and GSE246425 (younger vs. older OA cartilage). TPM-normalized expression matrices provided by GEO were used directly for downstream analyses. Pathway activity was quantified using single-sample gene set enrichment analysis (ssGSEA) implemented in the GSVA package in R, generating per-sample enrichment scores for gene sets related to the DISC–Caspase-8 axis, inflammation, and senescence. Senescence was assessed using the SAUL_SEN_MAYO gene set, and inflammatory signaling using the HALLMARK_INFLAMMATORY_RESPONSE gene set from MSigDB. Heatmaps were generated using the pheatmap package with Z-score–standardized TPM values. Group differences in ssGSEA scores were evaluated using two-sided independent-samples t-tests, and correlations between pathway scores were assessed using Pearson correlation analysis. Additional public transcriptomic datasets were analyzed to evaluate CASP8-related patterns in cartilage aging and experimental OA. The age-based human cartilage dataset GSE287861 was used to compare young and old cartilage samples, and the murine destabilization of the medial meniscus (DMM) dataset GSE26475 was used to compare sham-operated and DMM-operated samples at 6 h, 3 days, and 7 days after surgery. Group comparisons and correlation analyses were performed as described above, details are provided in the Supplementary Methods.

#### Single-cell RNA-seq analysis

Single-cell RNA-seq data from human OA- and non-OA articular cartilage were obtained from the Gene Expression Omnibus (GEO; GSE255460) and analyzed using Seurat (v5). Low-quality cells were excluded based on the number of detected genes (< 200 or > 6,000) and mitochondrial gene content (> 10%). Data were log-normalized, highly variable genes were identified, and scaled expression values were used for downstream analyses. Dimensionality reduction was performed using principal component analysis (PCA), followed by batch-effect correction using Harmony. Cells were clustered using a shared nearest-neighbor graph and visualized by uniform manifold approximation and projection (UMAP). Cell types were annotated based on established marker genes.

To characterize caspase-8–associated transcriptional programs at the single-cell level, pathway activity scores were calculated using UCell, a rank-based gene set scoring method optimized for single-cell RNA-seq data [18]. Caspase-8 signaling activity was assessed using either CASP8 gene expression or caspase-8 activation machinery score, defined based on key components involved in caspase-8 recruitment and activation, including *CASP8*,* FADD*,* TRADD*,* CASP10*.

Analyses were restricted to CASP8-positive or caspase-8 activation machinery–positive cells, defined as cells with expression values or UCell scores greater than zero. Within these positive cell populations, cells were further stratified into high and low groups based on the median CASP8 gene expression level or caspase-8 activation machinery score, respectively. Differential gene expression analyses were performed using the Wilcoxon rank-sum test implemented in Seurat (log2 fold-change threshold = 0.25; min.pct = 0.1). Genes with an adjusted *P* value < 0.05 and |log2 fold change| > 0.5 were considered statistically significantly regulated.

Functional enrichment analyses were conducted using both over-representation analysis (ORA) and gene set enrichment analysis (GSEA). Enrichment was assessed using Gene Ontology (GO) Biological Process (BP), KEGG, Reactome, Hallmark pathways, and the SAUL_SEN_MAYO senescence gene set. Results were visualized using dot plots, enrichment maps, and heatmaps where appropriate. Ligand–receptor-based cell–cell communication analysis was performed using the annotated single-cell dataset to compare inferred intercellular signaling between CASP8-high and CASP8-low chondrocyte populations across chondrocyte subtypes. Differential interaction number, communication strength, pathway-level information flow, and prioritized ligand–receptor pairs were summarized. Detailed filtering criteria, database settings, and analytical parameters are provided in the Supplementary Methods. As a sensitivity analysis, an in silico CASP8 knockout analysis usingscTenifoldKnk was performed within individual chondrocyte subpopulations. Genes predicted to be altered after CASP8 perturbation were subjected to functional enrichment analysis separately for each subpopulation using Metascape. Detailed preprocessing and analytical procedures are provided in the Supplementary Methods.

#### Spatial transcriptomic analysis

A publicly available spatial transcriptomic cartilage dataset (GSE254844) was analyzed to assess the zonal distribution of CASP8 expression across cartilage regions and loading conditions. Detailed preprocessing and analytical procedures are provided in the Supplementary Methods.

### Mendelian randomization (MR) analysis

We adopted a combinatorial Mendelian Randomization strategy, integrating two-sample MR and summary-data–based Mendelian Randomization (SMR), to investigate the causal role of CASP8 and related regulators in OA. In two-sample MR analyses, genetic variants associated with gene expression (eQTL), DNA methylation (mQTL), or splicing (sQTL) were used as instrumental variables. Causal effects were estimated using the inverse-variance weighted (IVW) method implemented in the TwoSampleMR package, with MR-Egger regression applied for sensitivity analysis to assess potential horizontal pleiotropy. Complementarily, SMR analysis was performed using the SMR Portal (https://yanglab.westlake.edu.cn/smr-portal/), integrating OA GWAS summary statistics with publicly available multi-tissue eQTL, sQTL, and mQTL datasets. The HeterogeneIty independent Instruments (HEIDI) test was used to distinguish putative causal or pleiotropic associations from signals driven by linkage disequilibrium. Significant SMR associations were defined by an SMR P value < 0.05, with a HEIDI P value > 0.01 required to support robust associations.

### Proteomics analysis

LC-MS/MS analysis was performed on an Orbitrap Astral mass spectrometer (ThermoFisher Scientific) in DIA mode, following the protocol previously established [16]. Briefly, proteins were extracted from chondrocytes treated with Z-IETD-FMK or vehicle control using a urea-based lysis buffer. After reduction, alkylation, and trypsin digestion, peptides were analyzed using a library-free DIA workflow. Differential expression and functional enrichment analyses were conducted as described previously [16]. To assess the potential structural compatibility between caspase-8 and selected downstream proteins altered after Z-IETD-FMK treatment, in silico protein–protein docking analysis was performed using proteomic candidates identified from the quantitative proteomics results. Predicted binding free energies and representative docking poses were summarized, with detailed methodological information provided in the Supplementary Methods.

### Statistical analysis

Statistical analyses were performed using GraphPad Prism (v10.2.3) and R (v4.2.2). Data are presented as medians with interquartile ranges (IQRs), unless otherwise noted. Changes in CASP8 expression were expressed as log₂ fold changes relative to untreated controls and evaluated using one-sample t-tests. Paired comparisons between matched experimental conditions (e.g., inhibitor vs. vehicle from the same donor) were assessed using paired t-tests. For experiments involving multiple treatments, one-way ANOVA with Holm–Sidak correction or two-way ANOVA with Geisser–Greenhouse adjustment followed by Šídák multiple-comparisons tests were applied, as appropriate. A two-sided P value < 0.05 was considered statistically significant.

## Results

### Transcriptomic analysis reveals transcriptional activation of the DISC–Caspase-8 axis in OA chondrocytes

**Fig. 2:**
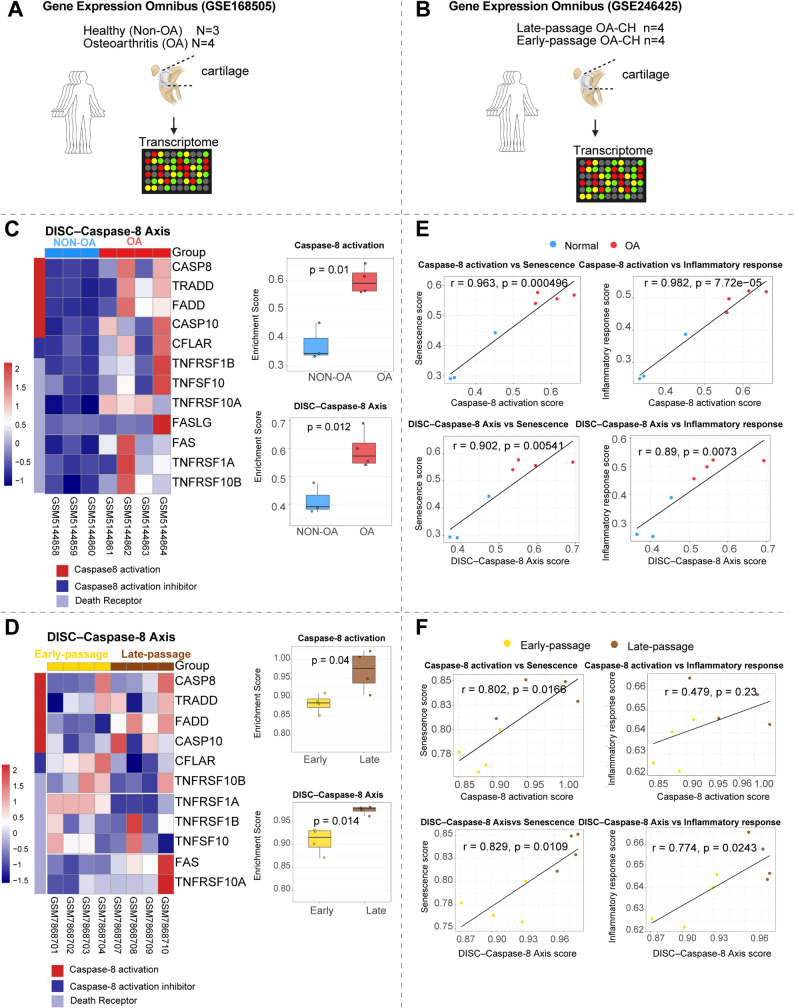
Transcriptomic Analysis reveals Activation of the DISC–Caspase-8 axis in OA Cartilage and senescent OA Chondrocytes. **A**–**B** Schematic overview of the transcriptomic datasets used in this study, including GSE168505 (non-OA- versus OA cartilage) and GSE246425, which compares early- and late-passage (senescent) OA chondrocytes representing non-senescent and senescent states in vitro. (**C**, **D**) Heatmaps showing the expression of genes involved in the death-inducing signaling complex (DISC)–Caspase-8 axis, including CASP8, TRADD, FADD, CASP10, CFLAR, TNFRSF1A/B, TNFRSF10A/B, and FAS/FASLG. Box plots compare enrichment scores for caspase-8 activation and DISC–Caspase-8 signaling between groups (unpaired t-test). **E**, **F** Correlation analyses between caspase-8 activation or DISC–Caspase-8 axis scores and gene signatures associated with cellular senescence and inflammatory responses. Pearson’s correlation coefficients (r) and corresponding P-values are indicated

To investigate the transcriptional activation pattern of caspase-8 and its upstream signaling components in OA chondrocytes, we analyzed two independent transcriptomic datasets from the Gene Expression Omnibus (GSE168505 and GSE246425). In dataset GSE168505, comparing OA and non-OA cartilage (Fig. 2A), we observed an upregulation of the caspase-8 activation signature and the “Death-Inducing Signaling Complex” (DISC) – Caspase-8 axis genes, including CASP8, TRADD, FADD, CASP10, CFLAR, and death receptors (TNFRSF1A/B, TNFRSF10A/B, FAS, FASLG – CD178) in OA samples (Fig. 2C). Both enrichment scores were significantly elevated in OA samples compared with non-OA chondrocytes (Fig. 2E). Correlation analysis further revealed that caspase-8 activation is strongly associated with senescence and inflammatory response signatures, and similar correlations were observed for the DISC–Caspase-8 axis suggesting that caspase-8 activation links inflammatory and degenerative signaling processes in OA chondrocytes.

To explore senescence-associated effects, we analyzed dataset GSE246425, comparing early-passage (P1–P3) and late-passage (P15–P18) OA chondrocytes (OA-CH), which represent non-senescent and replicative senescent states in vitro, respectively (Fig. 2B). Consistent with disease progression, both caspase-8 activation and the DISC–Caspase-8 axis scores were significantly elevated in late-passage, senescent OA chondrocytes (Fig. 2D). Correlation analysis further confirmed that caspase-8 activation and apoptotic DISC–Caspase-8 signaling remained tightly associated with cellular senescence (Fig. 2F).

To assess *CASP8*-related transcriptional changes in natural aging and experimental OA, we analyzed human aging (GSE287861) and murine DMM (GSE26475) datasets. In aged human cartilage, *CASP8* expression was significantly elevated and positively correlated with senescence scores, but not with inflammatory response scores. Furthermore, its upstream/downstream components and associated activation scores (including the DISC–Caspase-8 axis) showed no consistent increases (Suppl. Figure 2). Similarly, in the murine DMM model, neither *Casp8* expression nor its activation scores exhibited consistent changes compared to sham controls, and correlation analyses revealed no consistent associations with senescence or inflammatory phenotypes at any postoperative time point(Suppl. Figure 3).

Together, these results indicate that the robust transcriptional activation of the caspase-8/DISC axis—and its strong coupling to inflammatory and senescence networks—is a specific hallmark of osteoarthritic pathology and replicative senescence, distinct from the patterns observed in physiological aging or acute mechanical injury.

### Single-cell and spatial transcriptomic analysis reveals distinct distribution and functional bias of caspase-8 and DISC signaling in OA cartilage

**Fig. 3:**
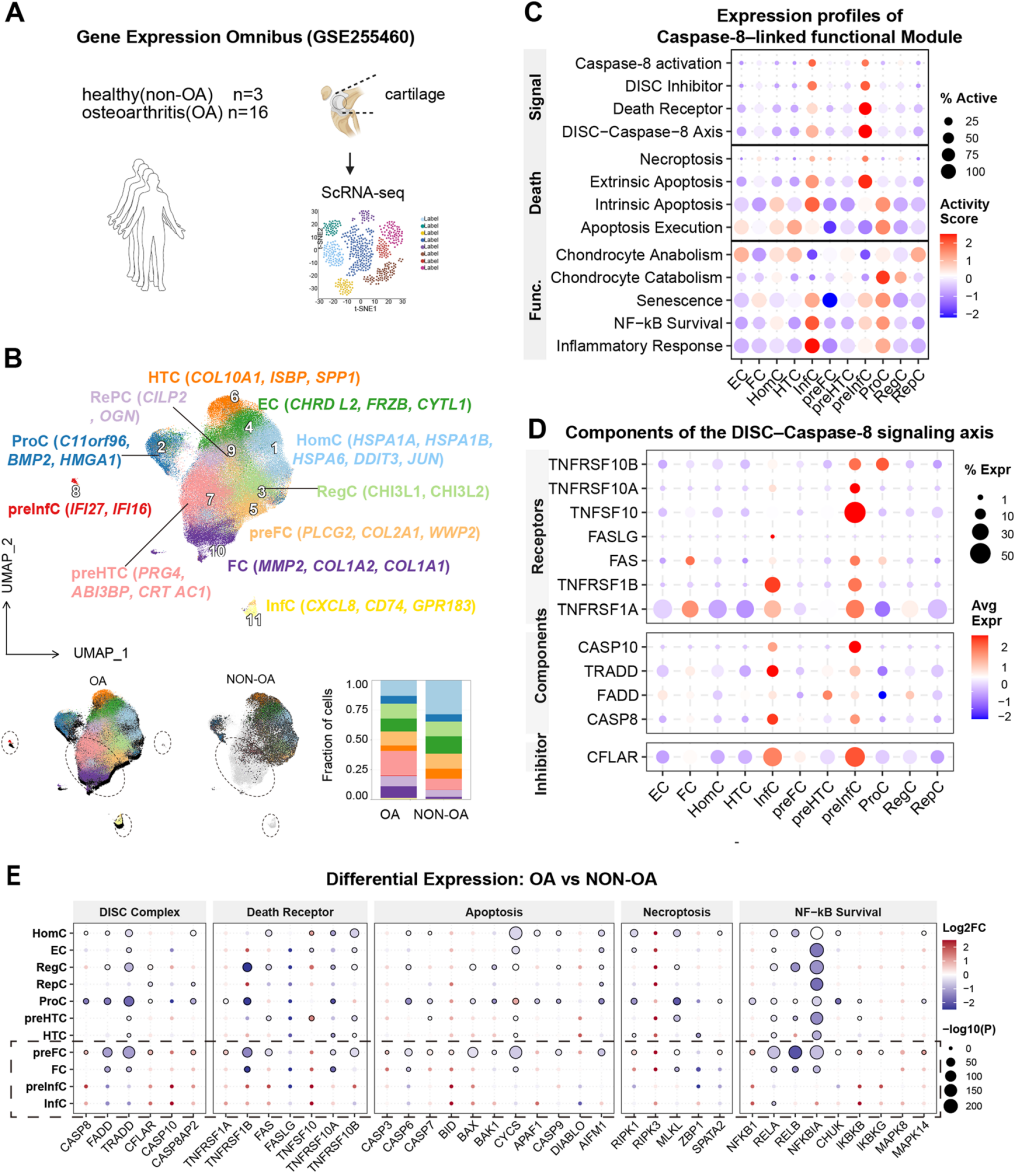
Single-Cell Profiling Reveals Lineage-Specific Enrichment of Caspase-8 in Inflammatory Subsets (**A**) Scheme of dataset GSE255460 (16 OA-, 3 non-OA cartilage samples) and analysis workflow. **B** UMAP plot showing 11 chondrocyte subtypes, including HomC, RegC, ProC, FC, HTC, preHTC, InfC, preInfC, EC, preFC and RePC. Bar plot compares relative fractions between OA- and non-OA cartilage, showing expansion of InfC/preInfC and FC with reduced HomC and RegC in OA samples. **C** Heatmap summarizing activity scores of caspase-8 - linked modules (e.g., DISC, apoptosis, senescence, NF-κB survival, inflammatory response). Detailed gene lists for each module are provided in Supplementary Table 2. **D** Expression patterns of DISC–Caspase-8 axis components (CASP8, FADD, TRADD, CFLAR, TNFRSF family) across chondrocyte subtypes. **E** Differential expression of DISC, death receptor, apoptotic, necroptotic, and NF-κB survival genes in OA- vs. non-OA chondrocytes

To elucidate the cellular heterogeneity and functional remodeling of caspase-8 signaling in OA, we analyzed single-cell RNA-sequencing (scRNA-seq) data from 16 OA- and 3 non-OA cartilage samples (GSE255460) (Fig. 3A).

Unsupervised clustering resolved multiple clusters, which were annotated according to the subpopulation definitions reported in the original study [19], resulting in 11 distinct chondrocyte subpopulations, including homeostatic chondrocytes (HomC), regulatory chondrocytes (RegC), proliferative chondrocytes (ProC), fibrocartilage chondrocytes (FC), pre-fibrocartilage chondrocytes (preFC), hypertrophic chondrocytes (HTC), pre-hypertrophic chondrocytes (preHTC), reparative chondrocytes (RepC), effector chondrocytes (EC), inflammatory chondrocytes (InfC), and pre-inflammatory chondrocytes (preInfC) (Fig. 3B). Comparative compositional analysis revealed pronounced perturbations in OA cartilage homeostasis. Inflammatory (InfC, preInfC), fibrocartilage-like (FC), and pre-hypertrophic (preHTC) populations were markedly expanded, whereas homeostatic (HomC) and hypertrophic (HTC) subsets were substantially depleted (Fig. 3B).

To characterize caspase-8 signaling across these cell subtypes, we mapped functional module activities associated with caspase-8 regulation (Fig. 3C). Caspase-8 activation and DISC signaling modules were preferentially enriched in inflammatory (InfC, preInfC) cell clusters, while HomC, FC and RegC cell populations exhibited comparatively low activity of these modules.

Moreover, extrinsic (pathway that starts outside the cell in the extracellular environment) apoptosis scores were distinctly enriched in inflammatory subsets (InfC, preInfC), indicating a specific activation pattern rather than a broad distribution across cell types. In parallel, NF-κB survival and inflammatory response modules showed the highest activity within the same clusters (InfC, preInfC), spatially overlapping with caspase-8 activation and extending partially to the proliferation subpopulation (ProC) (Fig. 3C).

At the molecular level, components of the DISC–Caspase-8 signaling axis showed distinct expression patterns across chondrocyte populations (Fig. 3D). Receptor genes (e.g., TNFRSF1A/B, TNFSF10), DISC components genes (TRADD, FADD, and CASP8), and the inhibitory regulator CFLAR were all highly expressed in inflammatory subsets (InfC and preInfC), whereas their expression levels were relatively low in other clusters. Notably, TNFRSF1A displayed a broader expression across multiple chondrocyte subpopulations (Fig. 3D).

In contrast to the bulk mRNA analysis showing an overall upregulation of the DISC–Caspase-8 activation signature in OA cartilage (Fig. 2C), cell type–specific differential expression analysis revealed a decoupled gene expression pattern between CASP8 and its DISC adaptor components across chondrocyte subpopulations comparing OA- with non-OA samples (Fig. 3E). The most pronounced differences were observed in OA-enriched pathological subsets, including FC-like (preFC/FC) and inflammation-associated (preInfC/InfC) chondrocytes. In FC-like chondrocytes, CASP8 and CASP10 exhibited concordant expression changes, with CASP8 increased in preFC cells, while CASP10 was less regulated but showed a similar direction of regulation. In contrast, the key DISC adaptor genes FADD and TRADD were significantly downregulated, accompanied by an upregulation of CFLAR. In these subsets, downstream executioners of apoptosis and necroptosis showed no consistent upregulation. Furthermore, NF-κB pathway components were largely reduced or unchanged in FC-like cells. In inflammation-associated chondrocytes, the upregulation of death receptors and CASP8/10 was not accompanied by increased DISC adaptors or execution genes. Instead, these cells exhibited elevated NF-κB and MAPK stress signaling.

For *CASP8* at the tissue level, we analyzed a spatial transcriptomic dataset (GSE254844) comprising control, non-weight-bearing (NWB), and weight-bearing (WB) cartilage. *CASP8* was detectable across all examined zones—including the articular surface (AS), superficial zone (SZ), middle zone (MZ), and deep zone (DZ)—but exhibited a zone- and loading-dependent distribution. Notably, the highest mean *CASP8* expression was observed in the AS under non-weight bearing (NWB) conditions, which was significantly higher than that in the weight-bearing (WB) group (*P < 0.05*). In contrast, CASP8 expression showed an opposite pattern in the deeper cartilage zones, including the SZ, MZ, and DZ. While expression in the NWB group decreased relative to the articular surface, the WB group showed relatively higher CASP8 transcript levels across these deeper regions (Suppl. Figure 4).

Collectively, these analyses indicate that CASP8 expression is enriched in OA-associated chondrocyte subsets and shows regional, loading-dependent heterogeneity across cartilage zones. However, this increase was not accompanied by coordinated regulation of core DISC adaptor components or downstream apoptotic execution genes, suggesting that CASP8 expression is decoupled from canonical DISC-mediated apoptotic signaling output in OA cartilage.

### CASP8-high chondrocytes exhibit suppressed matrix biosynthesis and enhanced inflammatory-fibrotic signaling

**Fig. 4:**
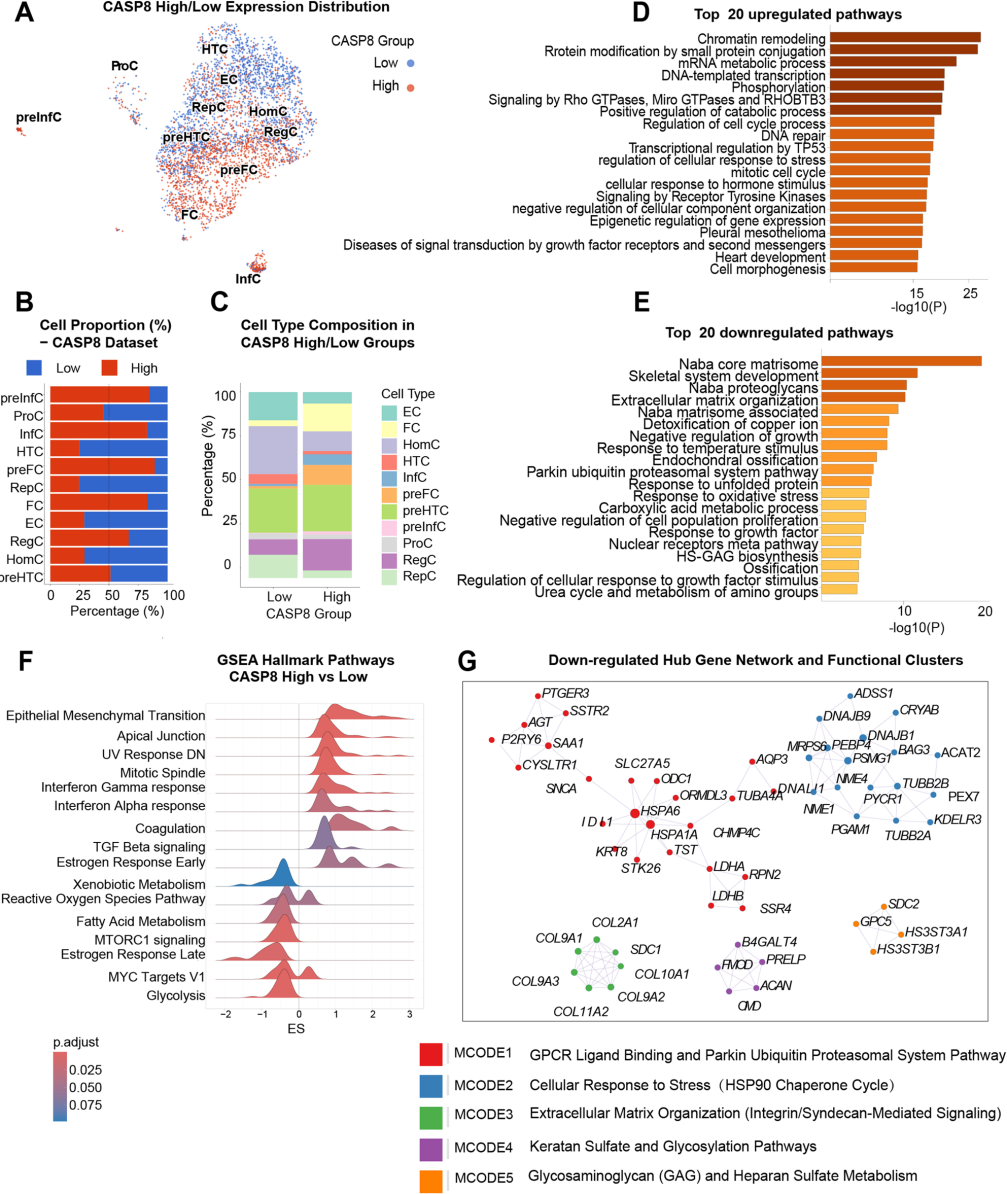
CASP8-High Expressing Chondrocytes Exhibit Suppressed Matrix Biosynthesis and Enhanced Inflammatory-Fibrotic Signaling. **A** UMAP plot showing CASP8 high and low gene expression distributions across chondrocyte subsets. **B** Cell-type-specific proportions of CASP8-high and CASP8-low groups. **C** Composition of chondrocyte subtypes within CASP8-high and CASP8-low expressing groups, revealing enrichment of inflammatory (InfC, preInfC), fibrocartilage-like (FC), regulatory (RegC) and pre-hypertrophic (preHTC) cell subpopulations in the CASP8 high-expression population. **D**–**E** Top 20 up- and down-regulated biological processes based on functional enrichment analysis. **F** Gene Set Enrichment Analysis (GSEA) of hallmark pathways showing enrichment of EMT, interferon responses, TGF-β/mTOR signaling, and glycolysis in CASP8-high cells. **G** Protein–protein interaction network of down-regulated hub genes, highlighting clustered modules related to extracellular matrix (ECM) organization, proteostasis, and glycosaminoglycan metabolism

To explore how high CASP8 gene expression reshapes cellular programs in OA, CASP8-positive chondrocytes were stratified into CASP8-high and CASP8-low gene expressing subgroups using the median CASP8 expression value as the cutoff. (Fig. 4A). CASP8-high cells were predominantly distributed within fibrocartilage-like (FC, preFC) and inflammatory (InfC, preInfC) clusters, whereas homeostatic (HomC), hypertrophic (HTC), effector (EC) and reparative (RepC) chondrocytes were enriched in the CASP8-low group (Fig. 4B–C).

Pathway enrichment analysis of differentially expressed genes between CASP8-high and CASP8-low chondrocytes revealed a clear functional divergence in transcriptional programs (Fig. 4D–E). In CASP8-high cells, upregulated pathways were predominantly enriched for processes related to catabolic processes and stress response, to chromatin remodeling, protein modification, DNA repair, TP53-associated transcriptional regulation, and Rho GTPase–associated signaling pathways, reflecting enhanced transcriptional and signaling activity (Fig. 4D). In contrast, pathways downregulated in CASP8-high chondrocytes were mainly associated with response to oxidative stress and growth factors, extracellular matrix (ECM) organization, proteoglycan biosynthesis, skeletal system development, and other matrix- and tissue-structural programs (Fig. 4E).

Gene set enrichment analysis (GSEA) further demonstrated that CASP8-high chondrocytes exhibited relative suppression of metabolic and biosynthetic pathways, including fatty acid metabolism, glycolysis, MTORC1 signaling, and reactive oxygen species–associated pathways. In contrast, inflammatory and fibrotic signaling programs, such as interferon-α/γ responses, TGF-β signaling, epithelial–mesenchymal transition (EMT), and coagulation, were positively enriched in CASP8-high cells (Fig. 4F).

Protein–protein interaction (PPI) analysis revealed that downregulated genes in CASP8-high chondrocytes highlighted coordinated suppression of proteostasis-related signaling, cellular stress responses, and the hyaline cartilage ECM phenotype (Fig. 4G). Specifically, these included GPCR ligand binding components and proteostasis-related genes (e.g. PTGER3, P2RY6, SNCA), stress-response chaperones (HSPA1A, HSPA6, DNAJB1), and key articular cartilage matrix genes (COL2A1, COL9A1/2/3, COL10A1, COL11A2, ACAN, SDC1), together with genes involved in proteoglycan and glycosaminoglycan biosynthesis (B4GALT4, HS3ST3A1, SDC2).

Together, these results delineate a coordinated transcriptional state in which elevated CASP8 gene expression is associated with enrichment of chromatin-associated and transcriptional regulatory programs, alongside interferon- and TGF-β–related inflammatory and fibrotic signaling, while concurrently suppressing hyaline cartilage matrix maintenance, cellular stress-adaptive capacity, and ECM homeostasis.

### Caspase-8 high expression defines distinct inflammatory, fibrotic, and metabolic programs across chondrocyte lineages

**Fig. 5:**
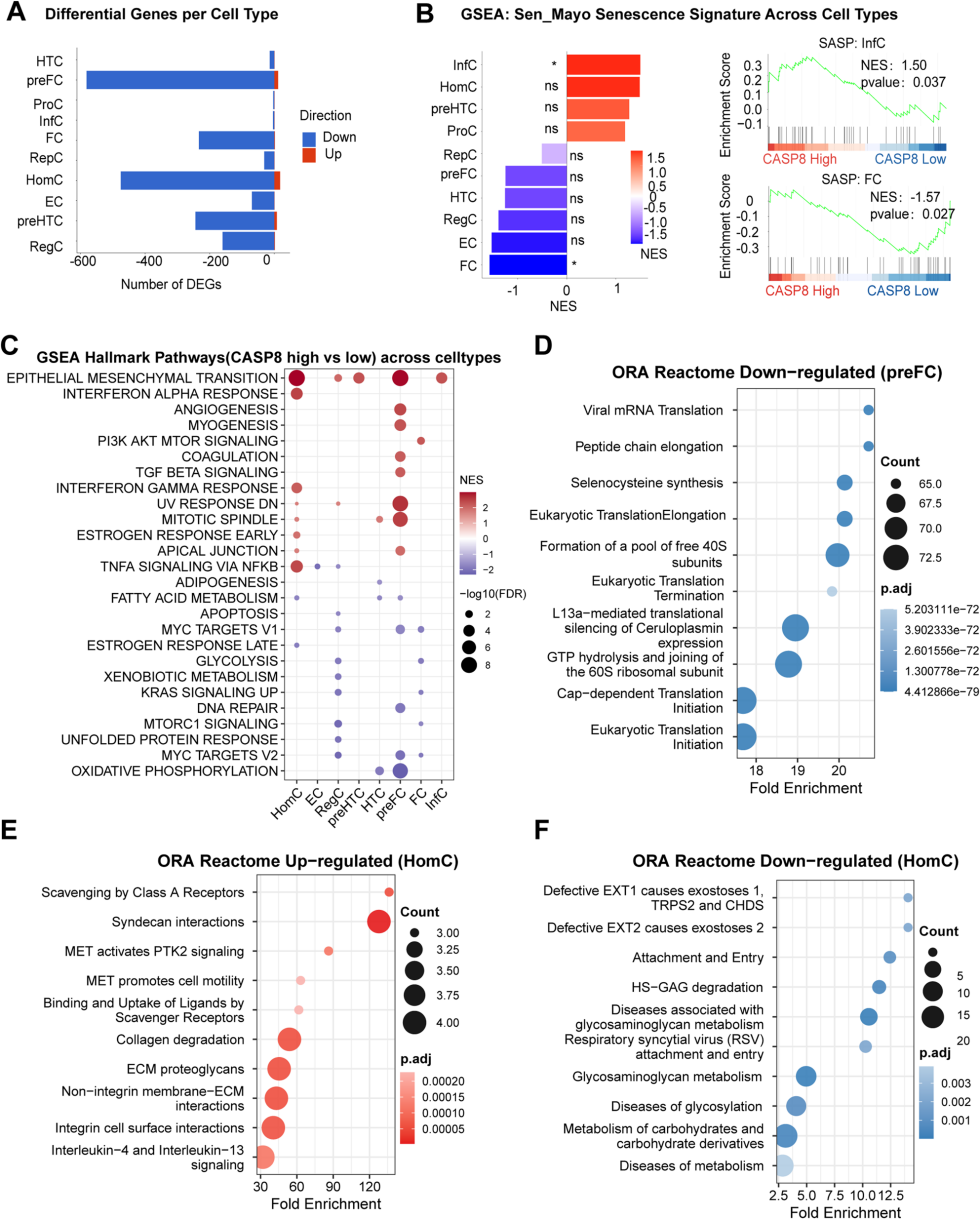
CASP8-High Expressing Chondrocytes Display Lineage-Specific Shifts Toward Pro-Inflammatory-, Fibrotic-, and Hypometabolic Phenotypes. **A** Number of up- and downregulated genes per chondrocyte subtype comparing CASP8-high and CASP8-low expressing groups. **B** Gene set enrichment analysis (GSEA) of the Sen_Mayo senescence signature showing enrichment of SASP programs in inflammatory chondrocytes (InfC) and suppression in fibrocartilage-like cells (FC). **C** GSEA Hallmark pathway enrichment across chondrocyte subtypes highlighting activation of interferon, TGF-β, EMT, and oxidative metabolism–related pathways in CASP8-high cells. **D** Downregulated ORA Reactome terms in pre-fibrocartilage (preFC) cells indicating suppression of translational and ribosomal functions. **E**, **F** ORA Reactome pathway enrichment in homeostatic chondrocytes (HomC) revealing activation of ECM proteolysis and interleukin signaling (**E**), alongside repression of glycosaminoglycan biosynthesis and metabolic processes **F**. Differential expression and enrichment analyses were performed with multiple-testing correction

To delineate the cellular consequences of high CASP8 gene expression across distinct chondrocyte lineages, we compared transcriptional programs between CASP8-high and CASP8-low cells in each subtype (Fig. 5A). Fibrocartilage-like chondrocytes (FC and preFC together) exhibited the largest number of differentially expressed genes, followed by homeostatic (HomC) cells, with most changes showing transcriptional downregulation across these populations.

Distinct senescence dynamics were observed among chondrocyte subtypes, with CASP8-high inflammatory cells (InfC) showing marked activation of SASP genes, whereas fibrocartilage-like cells (FC) exhibited significant suppression of the same program (Fig. 5B).

Gene set enrichment analysis (GSEA) revealed marked cell type–specific transcriptional programs associated with CASP8 expression (Fig. 5C). TNFα/NF-κB signaling was selectively enriched in CASP8-high homeostatic chondrocytes (HomC), whereas this pathway was not comparably activated in inflammatory (InfC) or fibrocartilage-like (preFC and FC) populations. By contrast, EMT was preferentially enriched in the inflammatory chondrocyte and PreFC subsets, while angiogenesis, and TGF-β signaling pathways were most prominent in pre-fibrocartilage (preFC) cells.

To further resolve subtype-specific transcriptional changes associated with CASP8 expression, over-representation analysis (ORA) was performed at the individual cluster level (Fig. 5D–F). In preFC chondrocytes, CASP8-high cells showed prominent downregulation of translation-related pathways, including cap-dependent translation initiation, ribosomal subunit assembly and peptide chain elongation (Fig. 5D). In contrast, HomC displayed enrichment of ECM remodeling and immune-related pathways, such as collagen degradation, syndecan–integrin interactions and interleukin-4/13 signaling (Fig. 5E), accompanied by reduced representation of pathways involved in glycosylation, glycosaminoglycan metabolism, glycosylation and carbohydrate derivative metabolism (Fig. 5F).

We next assessed inferred intercellular communication associated with CASP8 expression across chondrocyte subtypes. CASP8-high and CASP8-low populations showed differences in both interaction number and communication strength, with altered ligand–receptor patterns mainly involving adhesion-, extracellular matrix-, and growth factor-related interactions. Representative interactions included LGALS9–CD44, ITGA/ITGB-mediated interactions with collagens and FN1, COMP–collagen fibril interactions, and FGF-related ligand–receptor pairs, with prominent differences among fibrocartilage-like and inflammatory chondrocyte populations. (Suppl. Figure 5).

As a sensitivity analysis, we performed an in silico knockout of CASP8 within individual cell subpopulations and conducted enrichment analysis on the significantly altered genes, yielding similar results (Suppl. Figure 6).

Together, these results reveal pronounced subtype-specific transcriptional and intercellular communication programs associated with high CASP8 gene expression, characterized by enhanced senescence-associated signaling in inflammatory chondrocytes, selective inflammatory pathway activation in homeostatic chondrocytes, altered ligand–receptor communication involving ECM-, adhesion-, and growth factor-related signals, and coordinated suppression of metabolic and translational programs alongside pro-fibrotic and EMT-related signatures in pre-fibrocartilage cells.

### Caspase-8 activation amplifies inflammatory–senescence signaling and suppresses matrix homeostasis in OA chondrocytes

**Fig. 6:**
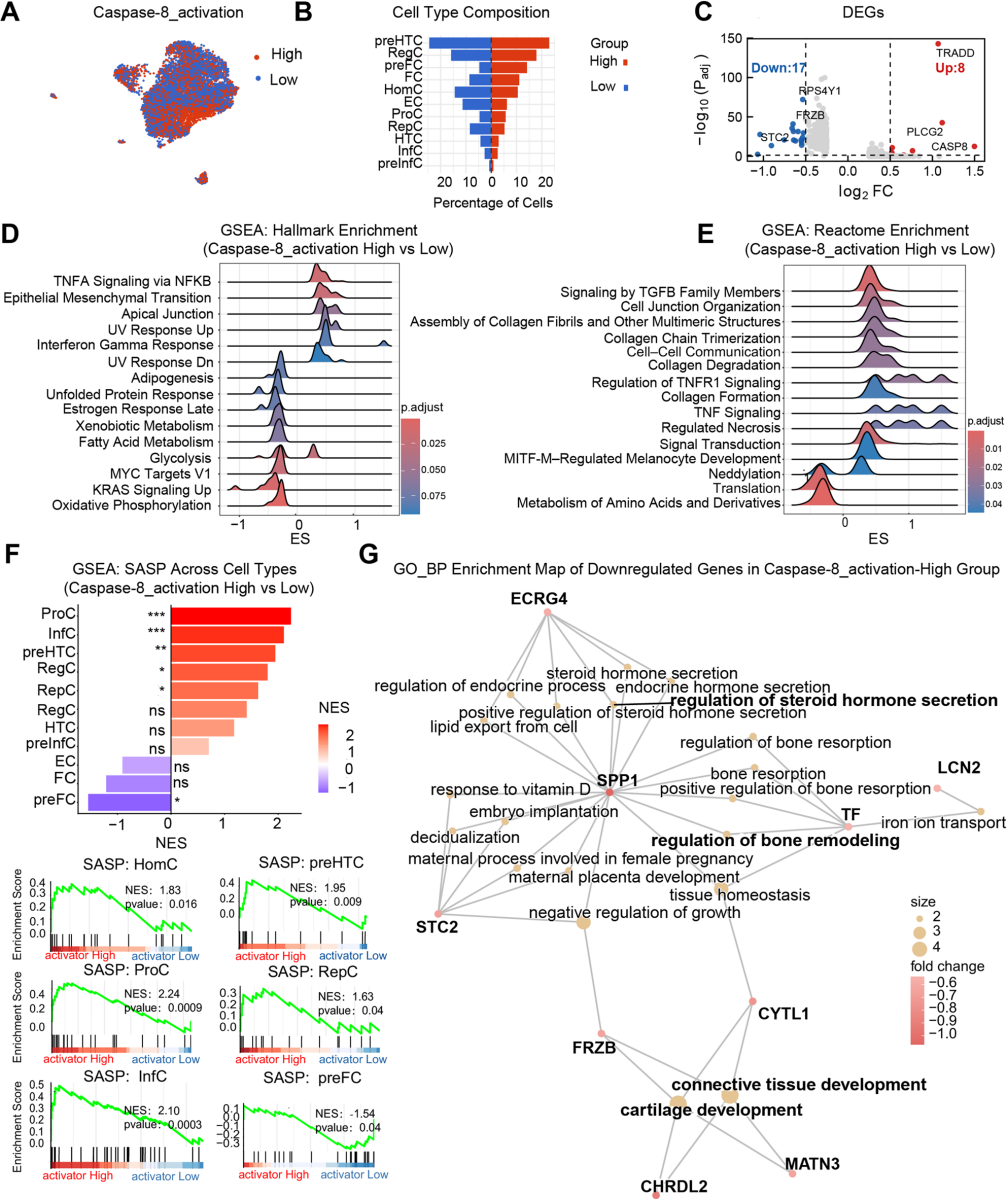
Caspase-8 Activation Amplifies Inflammatory, Senescence, and Fibrotic Signaling While Suppressing Matrix Homeostasis in OA Chondrocytes. **A** UMAP visualization of cells stratified by caspase-8 activation potential, calculated using UCell based scoring on core DISC components (CASP8, FADD, TRADD, CASP10). Cells were divided into high- and low expressing groups by median score. **B** Cell-type composition across caspase-8 activation states. **C** Differentially expressed genes between caspase-8 activation-high and -low cells. **D**, **E** GSEA showing enrichment of inflammatory and fibrotic pathways, including TNF-α/NF-κB, interferon, TGF-β, and EMT signaling. **F** GSEA of the Sen_Mayo signature indicating SASP enrichment across ProC, InfC, and RegC subsets. **G** GO enrichment of downregulated genes highlighting suppression of steroid hormone signaling, cartilage development, and ECM organization

Building on the identified transcriptomic features of CASP8-high cells, we next examined how caspase-8 activation potential relates to chondrocyte lineage distribution. Compared with CASP8–high cells (Fig. 4B–C), caspase-8 activation–high cells (cell populations with a high activation state of caspase-8) showed a more evenly distributed representation across chondrocyte subtypes, while still displaying relative enrichment within fibrocartilage-like populations, particularly preFC and FC cells (Fig. 6A–B).

At the molecular level, caspase-8 activation–high expression pattern recapitulated the inflammatory–fibrotic transcriptional profile observed in CASP8-high cells and further magnified senescence-associated alterations. CASP8 and its adaptor TRADD were significantly upregulated, whereas genes linked to chondrocyte anabolic activity and cartilage homeostasis, including STC2, FRZB, and RSPO4, were downregulated (Fig. 6C), indicating a decline in anabolic capacity.

Hallmark GSEA further revealed coordinated activation of TNFα/NF-κB, interferon-α/γ, TGF-β, and EMT signaling pathways in caspase-8 activation–high chondrocytes (Fig. 6D).

Reactome GSEA further highlighted TGFB-family signaling and collagen remodeling as the dominant features of caspase-8 activation–high cells, with enrichment of collagen trimerization/formation and degradation programs alongside cell-junction and cell–cell communication pathways (Fig. 6E).

Notably, cells with high caspase-8 activation potential displayed broader and stronger induction of the SASP phenotype compared with CASP8-high cells, spanning proliferation (ProC), inflammatory (InfC), and regulatory (RegC) subpopulations, whereas pre-fibrocartilage cells showed a significant suppression of the SASP program (Fig. 6F). Concurrently, downregulated genes in caspase-8 activation–high cells were enriched for steroid and hormone regulation, bone remodeling, and cartilage development pathways (Fig. 6G), suggesting a coordinated repression of differentiation- and tissue-maintenance programs.

Collectively, these data demonstrate that caspase-8 activation potential aligns inflammatory, senescence, and fibrotic transcriptional responses with repression of matrix anabolism in chondrocytes.

### Modification of caspase-8 catalytic activity modulates chondrocyte senescence, proliferation, migration, and matrix metabolism in OA chondrocytes

To validate the functional relevance of caspase-8 in OA pathology, primary OA- and non-OA chondrocytes were stimulated with TNF-α and/or treated with the caspase-8 inhibitor Z-IETD-FMK.

Overall, the 2D monolayer cultures displayed consistent and reproducible regulatory patterns whereas in 3D pellet micromass cultures mostly no uniform directions of regulation was detected (Suppl. Figure 1). For that we continued all further experiments in 2D monolayer culture only.

**Fig. 7:**
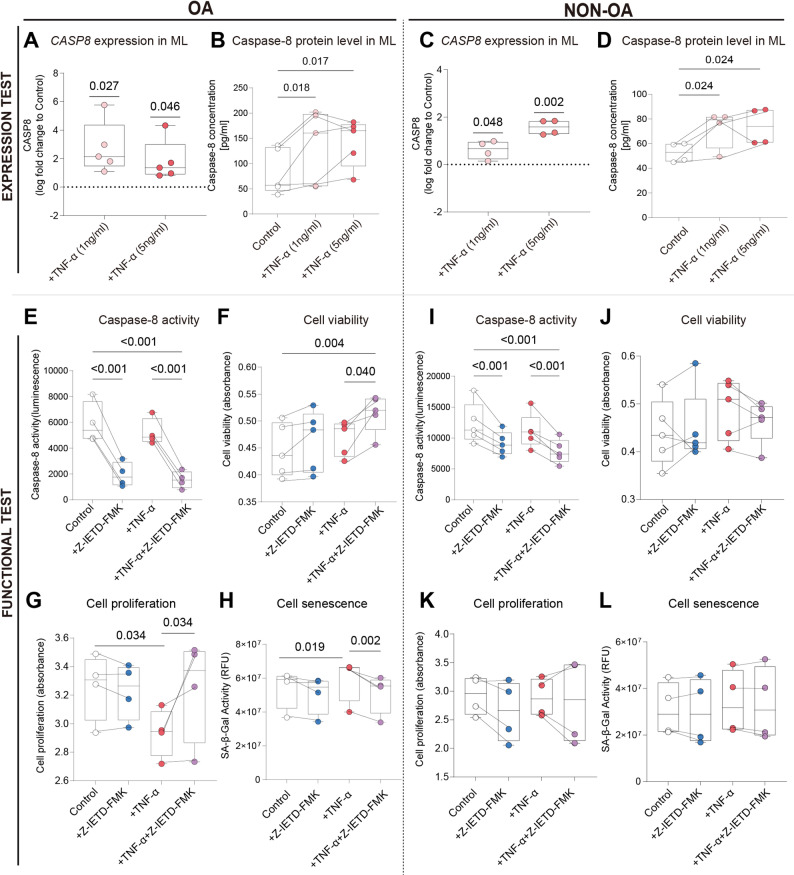
Modification of Caspase-8 Catalytic Activity Modulates Senescence, Proliferation, Migration, and Matrix Metabolism in OA Chondrocytes. **A**–**D** CASP8 mRNA expression and caspase-8 protein levels in OA (**A**, **B**) and non-OA (**C**, **D**) chondrocytes following TNF-α stimulation. **E**–**H** Functional assays in OA chondrocytes including determination of caspase-8 activity, viability, proliferation, and SA-β-gal activity, under basal conditions, TNF-α stimulation, caspase-8 inhibition (Z-IETD-FMK), or combined treatment. **I**–**L** Corresponding measurements in non-OA chondrocytes. Data are shown as median with interquartile range from independent donors. Statistical significance was determined using one-sample t-tests for mRNA expression (relative to control), paired t-tests for protein comparisons, and one-way ANOVA with Holm–Sidak correction for functional assays. *N* = 4–5

In OA chondrocytes, TNF-α stimulation (both concentrations) increased CASP8 mRNA and intracellular protein expression but did not significantly alter caspase-8 enzymatic activity (Fig. 7A, B, E). Treatment with the caspase-8 inhibitor Z-IETD-FMK significantly suppressed caspase-8 activity under both basal (no TNF-α) and TNF-α–stimulated conditions, confirming effective catalytic inhibition (Fig. 7E). Caspase activity inhibition, induced metabolic activity (cell viability) under inflammatory stimulation (+ TNF-α), and proliferation also showed a recovery in the presence of TNF-α (Fig. 7F, G). Notably, Z-IETD-FMK treatment markedly reduced senescence-associated (SA)-β-Galactose activity under both basal and TNF-α–stimulated conditions, indicating attenuation of cellular senescence (Fig. 7H). Apoptosis, indicated by induction of caspase-3/7 activity and Annexin V / 7-AAD staining was not induced by TNF-α stimulation-regardless of the concentration (1 ng/ml and 5ng/ml)- of chondrocytes up to 24 h (Suppl. Figure 7). Knocking down CASP8 genetically by using specific siRNA, we obtained an 85% knockdown in OA chondrocytes compared to non target(nt)-controls (Suppl. Figure 8) with no subsequent changes in cell senescence, proliferation, viability and apoptosis with and without TNF-α stimulation (Suppl. Figure 9).

**Fig. 8:**
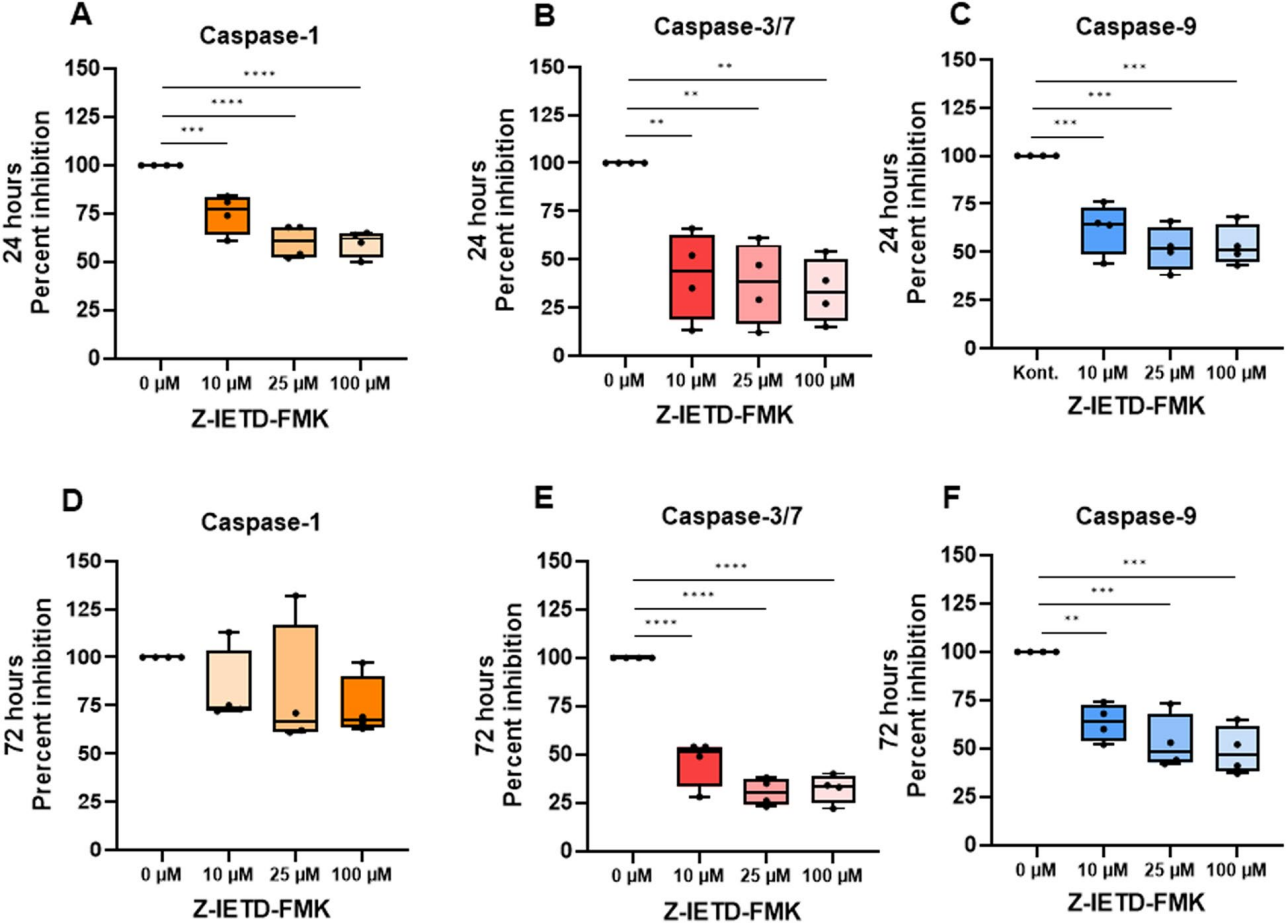
Activity of Caspase-1, Caspases-3/7 and Caspase-9 in the presence of Caspase-8 Inhibitor Z-IETD-FMK. OA chondrocytes were treated with caspase-8 inhibitor Z-IETD-FMK in concentrations from 0 μm to 100 µM for 24 (**A**-**C**) and 72 (**D**-**F**) hours. Subsequently, caspase-1 (**A**, **D**), caspases-3/7 (**B**, **E**) and caspase-9 (**C**, **F**) activities were measured with the corresponding Caspase- Glo^®^ luminescent assays. Each dot represents a donor. The statistical significance was determined using an Ordinary one-way-ANOVA test followed by Holm-Šídák’s multiple comparisons test. *p < 0.05; **p < 0.01; ***p < 0.001; ****p < 0.0001; *N* = 4

In non-OA chondrocytes, caspase-8 expression was induced by TNF-α both on mRNA and protein level (Fig. 7C, D). Caspase-8 activity was likewise suppressed by Z-IETD-FMK under both basal and TNF-α–stimulated conditions (Fig. 7I), but no significant changes were observed in cell viability, proliferation or SA-β-Gal across all treatment groups (*p* > 0.05) (Fig. 7J–L). Titration of chondrocytes with Z-IETD-FMK, using inhibitor time points at 12, 24, 48 and 72 h and inhibitor concentrations of 10, 25, 50 and 100 µM, revealed no differences between concentrations at the time points of 12 h and 72 h on caspase-8 activity. Incubation time points of 24 h and 48 h showed the strongest effect on reduction of caspase-8 activity when using the inhibitor concentration of 100 µM (Suppl. Figure 10). Analysis of activity of other caspases, e.g. caspase-1, caspase-3/7 and caspase-9 after treatment of OA chondrocytes with caspase-8 inhibitor Z-IETD-FMK using above tested different concentrations and timepoints revealed significant reduction of activity of all three caspases with all concentrations and under all time points (except for caspase-1 at the 72 h time point) (Fig. 8 and Suppl. Figure 11).

**Fig. 9:**
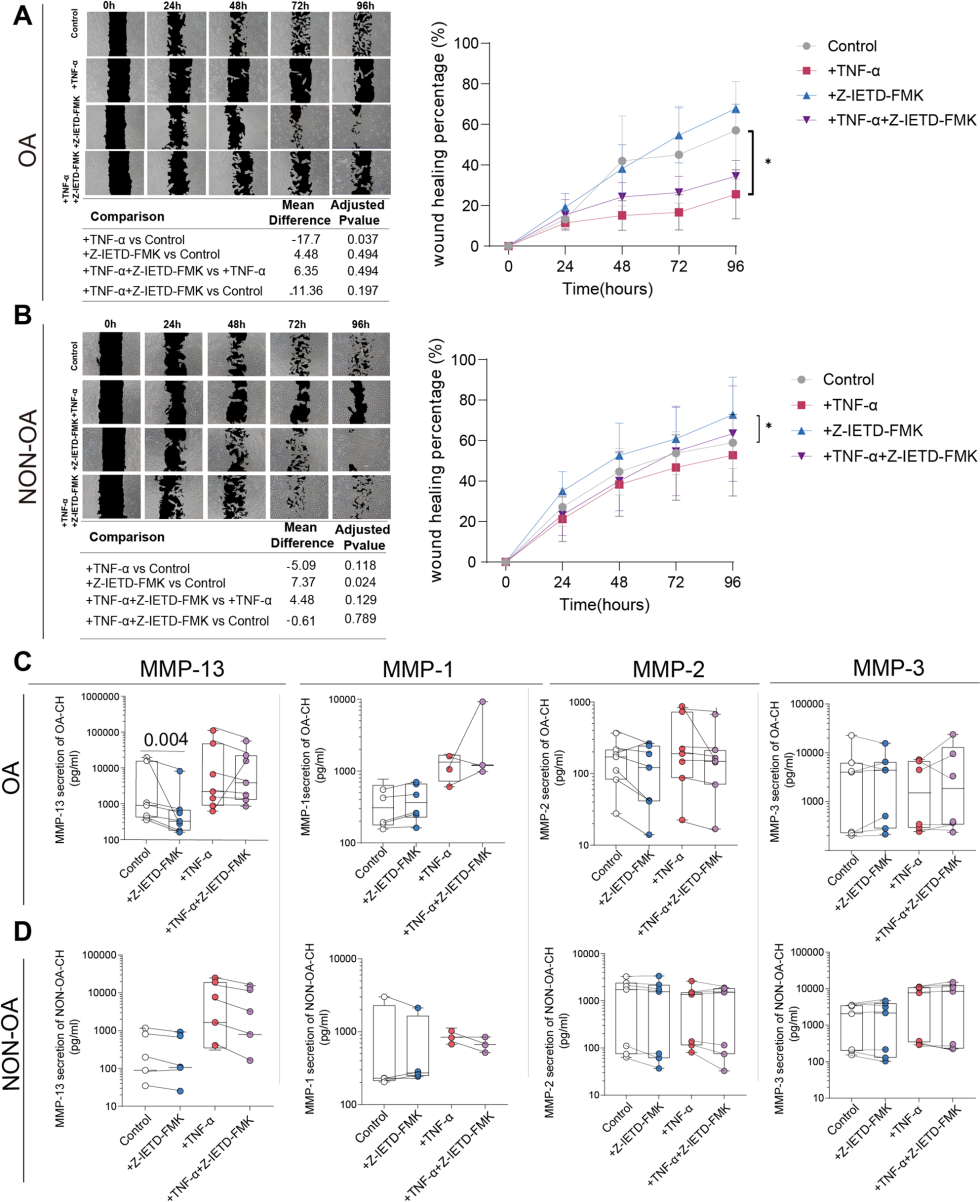
Inhibition of Caspase-8 Catalytic Activity Restores Gap Closing Capacity and Attenuates MMP-13 Secretion in OA Chondrocytes. **A** Representative images and quantitative analysis of wound (gap) closure (applying a scratch assay) in OA chondrocytes treated with TNF-α (1 ng/mL) and/or Z-IETD-FMK (100 µM) over 96 h. **B** Quantification of wound (gap) closure in non-OA chondrocytes under the same experimental conditions. **C** Concentrations of secreted MMP-1, MMP-2, MMP-3, and MMP-13 in culture supernatants of OA chondrocytes, measured by multiplex immunoassay or ELISA. **D** Corresponding MMP concentrations in supernatants of non-OA chondrocytes. Data are presented as median with interquartile range (IQR). Statistical comparisons for wound closure assays were performed using one-way ANOVA with Holm–Šídák correction. For MMP secretion assays, data were normalized within each experimental batch prior to statistical analysis and analyzed using two-way ANOVA with Geisser–Greenhouse correction followed by Šídák’s multiple comparisons test. *N* = 3–7

In wound-healing (migration) assays, OA chondrocytes displayed impaired gap closure capacity under TNF-α stimulation, which was partially rescued by Z-IETD-FMK treatment (Fig. 9A). In non-OA chondrocytes, Z-IETD-FMK significantly enhanced migration under basal conditions, whereas TNF-α alone or in combination with the inhibitor did not further alter wound closure speed (*p* > 0.1) (Fig. 9B).

Consistent with these functional improvements, Z-IETD-FMK treatment alone significantly reduced MMP-13 secretion in OA chondrocytes but not when co-stimulated with TNF-α or in non-OA chondrocytes, while levels of MMP-1, MMP-2, and MMP-3 remained unchanged in both cell types (*p* > 0.05) (Fig. 9C). In non-OA chondrocytes, no significant effects of the Z-IETD-FMK inhibitor on any of the MMP isoforms were observed (all *p* > 0.05) (Fig. 9D).

Taken together, these data demonstrate that usage of the caspase-8 inhibitor Z-IETD-FMK suppresses caspase-8 and downstream acting caspases enzymatic activity under both basal and inflammatory conditions, selectively alleviates TNF-α–induced senescence, and restores proliferation and migration while reducing MMP-13 production/secretion in OA chondrocytes, with minimal effects only in non-OA cells.

### Integrated proteomic analysis reveals apoptosis-independent suppression of inflammatory and senescence pathways after caspase-8 inhibition

#### PPI analysis

**Fig. 10:**
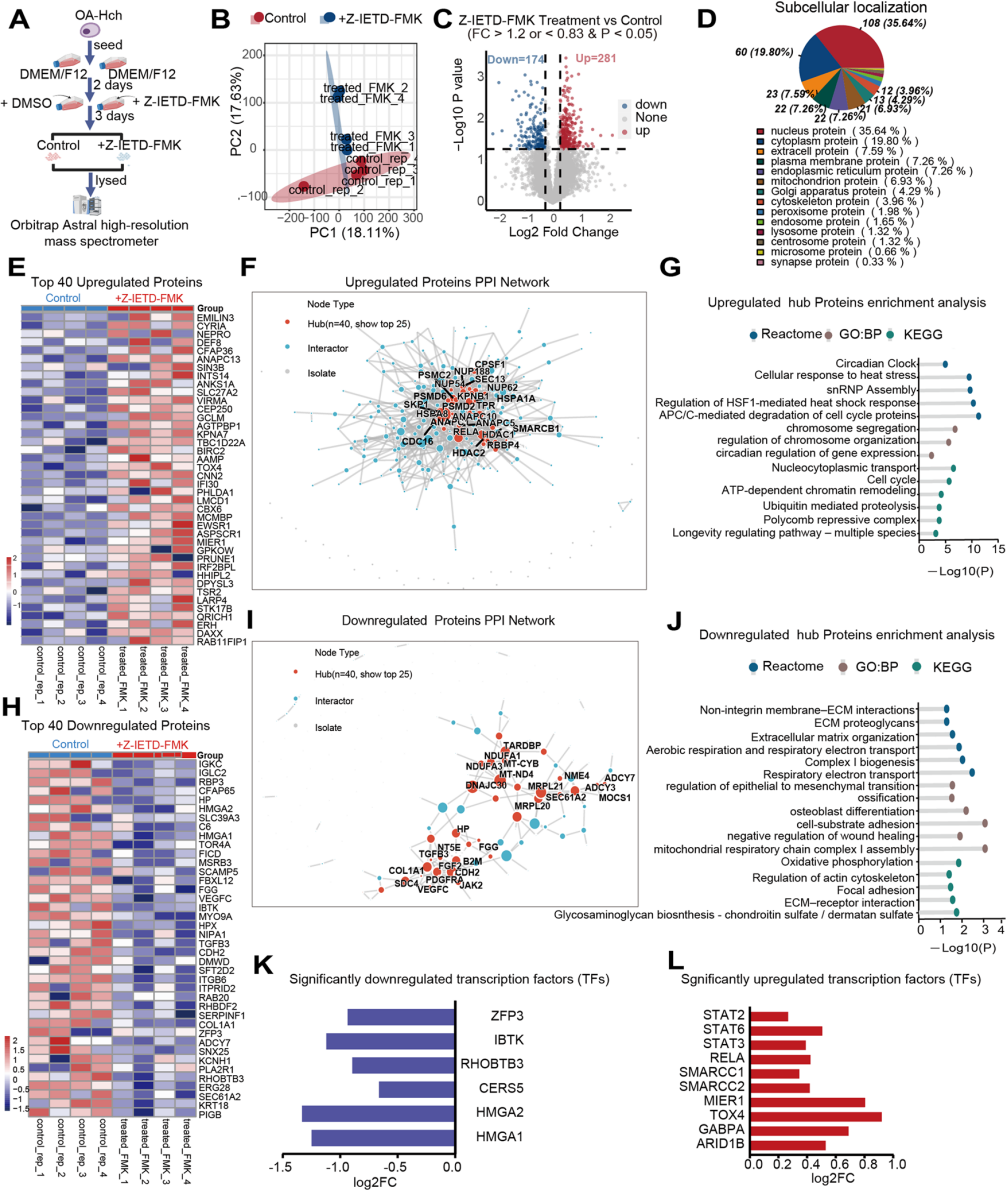
Caspase-8 Inhibition Reconfigures the Chondrocyte Proteome by Promoting Chromatin and Stress Response Mechanisms While Repressing Fibrotic Matrix Programs. **A** Experimental workflow for LC–MS/MS proteomic profiling following Z-IETD-FMK treatment. **B** Principle Component analysis (PCA) showing separation between inhibitor-treated and control samples. **C** Differentially expressed proteins. **D** Subcellular localization of regulated proteins. **E**, **H** Heatmaps of top up- and downregulated proteins. **F**, **I** Protein–protein interaction networks highlighting proteostasis/chromatin remodeling hubs (**F**) and fibrocartilage/mitochondrial modules (**I)**. **G**, **J** Functional enrichment of regulated proteins. **K**, **L** Predicted transcription factor activity

To define the molecular mechanisms underlying caspase-8 inhibition in OA chondrocytes, we performed high-resolution quantitative proteomics following Z-IETD-FMK treatment (Fig. 10A). Principal component analysis (PCA) showed distinct separation between treatment and control groups (Fig. 10B). A total of 281 proteins were up-regulated and 174 were down-regulated (Fig. 10C), mainly localized in the nucleus and cytoplasm (Fig. 10D).

Up-regulated proteins after caspase-8 inhibition (Fig. 10E) included EMILIN3, CYRIA, DEF8, and BIRC2, predominantly involved in cytoskeletal organization, nuclear regulation, and cellular stress adaptation.

Network integration (Fig. 10F) revealed a densely connected proteostasis–chromatin remodeling hub, comprising proteasome regulatory subunits (PSMD2, PSMD6, PSMC2), molecular chaperones (HSPA1A, HSPA8), and chromatin-associated factors (HDAC1/2, RBBP4, SMARCB1). These nodes were linked through ANAPC and nucleoporin (NUP) complexes, indicating an integrated network coupling protein quality control, transcriptional regulation, and nucleocytoplasmic transport. Functional enrichment (Fig. 10G) confirmed significant activation of pathways related to chromatin remodeling, cell-cycle control, and stress-response signaling, suggesting that caspase-8 inhibition promotes broad proteostatic and epigenetic reprogramming rather than apoptosis inhibition in OA chondrocytes.

In contrast, down-regulated proteins after caspase-8 inhibition (Fig. 10H), including VEGFC, TGFB3, COL1A1, HMGA1, and HMGA2, reflected suppression of fibrocartilage-like matrix remodeling and transcriptional activation.

Network analysis (Fig. 10I) revealed two major coordinated hub modules:

 [1] a fibrocartilage-like remodeling module centered on COL1A1, TGFB3, FGF2, PDGFRA, VEGFC, and SDC4, indicating attenuation of ECM restructuring and fibrotic remodeling; and

 [2] a mitochondrial–metabolic module encompassing MRPL21, NDUFA1, MT-CYB, DNAJC30, and TARDPB, signifying repression of oxidative phosphorylation and mitochondrial translation.

Consistent with these hub patterns, enrichment analysis (Fig. 10J) highlighted coordinated downregulation of ECM organization, cell adhesion, oxidative phosphorylation, and wound-healing–related pathways, suggesting a shift from energy-intensive fibrocartilage-like activation toward a metabolically adaptive, low-energy state. Finally, transcription factor analysis (Fig. 10K, L) revealed upregulation of STAT2, STAT3, STAT6, and chromatin regulators SMARCC1/2, accompanied by downregulation of HMGA1/2 and IBTK, indicating that caspase-8 inhibition reconfigures the transcriptional landscape via STAT- and chromatin-dependent remodeling rather than canonical apoptotic signaling.

To assess whether proteins perturbed after caspase-8 inhibition may have potential structural compatibility with caspase-8, we performed computational molecular docking using selected proteomic candidates. Favorable predicted binding free energies were observed for several proteins, including HDAC1, TGF-β1, PDGFRA, MMP13, HDAC2, RELA, TGF-β3, HSPA1A, JAK2, and FGF2, with HDAC1, TGF-β1, PDGFRA, and MMP13 showing relatively stronger predicted affinities (Suppl. Figure 12).

#### Pathway level analysis

**Fig. 11:**
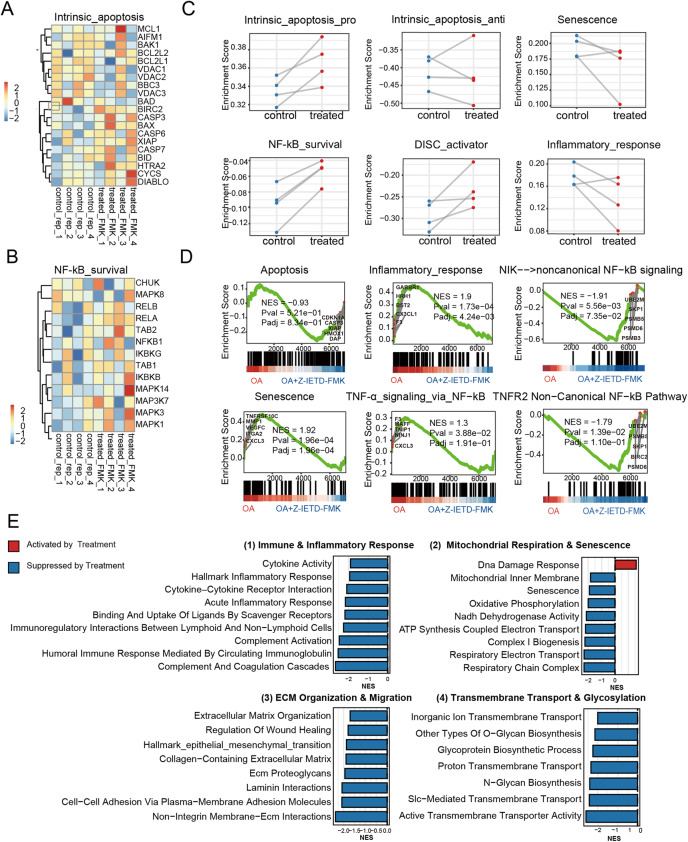
Caspase-8 Inhibition Suppresses Senescence- and Inflammation-associated Pathways at the Proteomic Level. **A**, **B** Protein abundance of intrinsic apoptosis regulators and NF-κB survival components. **C** Module-level enrichment scores showing reduced senescence and inflammatory signaling after caspase-8 inhibition. **D** Representative GSEA plots of suppressed or unchanged pathways. **E** Summary of pathway-level shifts following caspase-8 inhibition, showing Z-IETD-FMK t reatment vs. Control GSEA enrichment (all Padj<0.05)

Pathway-level analyses of global protein alterations after caspase-8 inhibition further revealed that intrinsic apoptosis remained largely unaltered, whereas inflammatory response, cellular senescence, and canonical NF-κB signaling were markedly suppressed. In parallel, non-canonical NF-κB survival modules displayed some enhancement in the presence of Z-IETD-FMK (Fig. 11).

At the individual protein level, intrinsic apoptosis regulators did not exhibit coordinated large-scale alterations (Fig. 11A). Both pro-apoptotic molecules (e.g., BAX, BID, CYCS) and anti-apoptotic proteins (e.g., BCL2L1, BCL2A1) showed modest, sample-dependent fluctuations without a consistent directional shift. Within the NF-κB survival module (Fig. 11B), most components remained largely stable across conditions, with only subtle upward tendencies for CHUK, RELA, and RELB in Z-IETD-FMK–treated cells, while TAB2, IKBKB, and MAPK1/MAP3K7 remained unchanged or showed only minor variability.

Sample-wise pathway scoring did not reveal statistically significant differences between both groups; however, clear directional tendencies were evident. Senescence and inflammatory-response scores mostly consistently shifted downward, whereas pro-apoptotic intrinsic apoptosis and NF-κB survival scores did not decrease and even showed upward trends after caspase-8 inhibition (Fig. 11C). These trends were subsequently validated by global proteome–based gene-set enrichment analysis (GSEA), which highlighted the suppression of inflammatory and senescence signatures, together with a shift from canonical NF-κB activation toward non-canonical NF-κB survival signaling, while apoptosis exhibited no significant changes (Fig. 11D).

Integrated GSEA further confirmed down-regulation of almost all investigated pathways, e.g. immune and inflammatory cascades, complement activation, including cytokine–cytokine receptor interaction and TNF-α/NF-κB pathways, together with reduced senescence signatures (Fig. 11E). Modules associated with ECM organization, wound-healing–related fibrotic remodeling were also attenuated, whereas only DNA damage responses exhibited activation.

Taken together, the proteomic evidence demonstrates that caspase-8 inhibition drives a coordinated, apoptosis-independent shift away from catabolic inflammatory and senescence-associated signaling, accompanied by secondary modulation of ECM/fibrotic remodeling and metabolic pathways in OA chondrocytes.

### Population-level genetic analysis supports CASP8 as a causal regulator in OA with broad tissue-wide effects

**Fig. 12:**
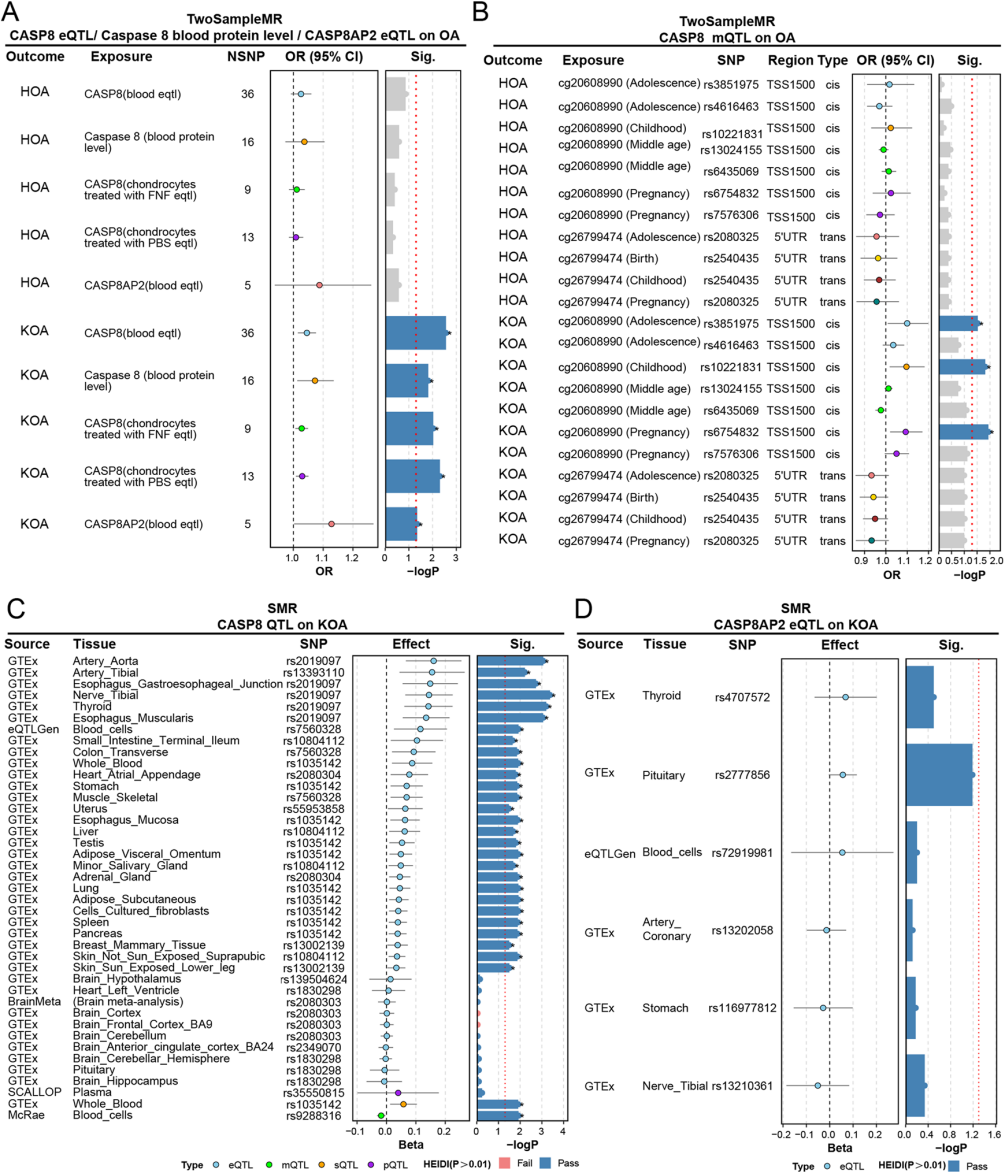
Genetic Causal Inference Establishes *CASP8* as a Risk Factor for OA Susceptibility with Systemic Regulatory Effects. **A** Two-sample Mendelian Randomization (MR) analysis (Inverse Variance Weighted method) showing the causal association between genetically predicted *CASP8* gene expression or circulating caspase-8 protein levels and OA risk in knee and hip cohorts. **B** Methylation QTL (mQTL)–based MR analysis using CpG sites within the *CASP8* promoter region (e.g., cg20608990) as instrumental variables. **C** Summary-data-based Mendelian Randomization (SMR) results for *CASP8* across multiple tissues, indicating the presence of colocalized eQTL and GWAS signals (HEIDI test *P* > 0.01). **D** SMR analysis of the caspase-8 adaptor gene *CASP8AP2*, showing no significant causal association, highlighting the specificity of the *CASP8* locus. Error bars indicate 95% confidence intervals. *P* < 0.05 indicates statistical significance. SNP= single nucleotide polymorphism; NSNP= number of SNPs. KOA= Knee OA; HOA = Hip OA

To validate the causal relevance of caspase-8 in OA at the population level, we performed two-sample Mendelian randomization (MR) and data-based SMR analyses using expression, methylation, and protein expression quantitative trait loci (eQTL, mQTL, and pQTL) as instrumental variables. These analyses consistently supported a causal role of CASP8 in OA (Fig. 12).

MR revealed that genetically predicted higher CASP8 expression, circulating caspase-8 protein levels, and CASP8AP2 gene expression (Caspase-8–associated protein 2, a scaffold protein involved in regulating caspase-8 recruitment and activation) were significantly associated with an increased risk of knee OA (KOA), whereas estimates for hip OA (HOA) followed the same positive direction but with weaker effect sizes (Fig. 12A). Causal estimates derived from eQTLs in chondrocytes and peripheral blood were concordant, supporting a positive regulatory direction for CASP8.

MR using CpG methylation QTLs (mQTLs) around the CASP8 promoter further corroborated this regulatory pattern. Three independent mQTL instruments derived from cg20608990 (TSS1500 region) demonstrated that lower methylation was causally associated with a higher risk of KOA (Fig. 12B). Although additional instruments and HOA estimates did not reach statistical significance, they pointed in the same risk-increasing direction.

Summary of SMR further confirmed the association of CASP8 with KOA across multiple tissues (Fig. 12C). The strongest colocalized signals were detected in subcutaneous and visceral adipose tissue, whole blood, skeletal muscle, and nerve and arterial tibial tissues, suggesting a systemic regulatory influence of caspase-8 beyond cartilage. In contrast, CASP8AP2 did not exhibit significant effects (Fig. 12D), indicating potential regulatory divergence between CASP8 and its adaptor gene.

Data sources, instrument SNPs, and sensitivity analyses are provided in Supplementary Table 1.

Collectively, these genetic data identify the CASP8 gene as a causal mediator of OA susceptibility, while revealing substantial tissue- and context-dependent heterogeneity, potentially arising from differential regulatory architecture across developmental and epigenetic contexts.

## Discussion

In this study, we identified caspase-8 as a central non-apoptotic regulator that integrates inflammatory, senescent, and fibrotic signaling in OA pathology. By combining bulk and single-cell transcriptomics, proteomics, functional assays, and Mendelian randomization, we delineate the caspase-8 expression pattern in OA pathology and provide genetic evidence for its causal contribution to knee OA risk. Together, these data support a model in which caspase-8 functions less as a classical apoptosis executor and more as a signaling hub that locks chondrocytes into a chronic inflammatory, maladaptive state. Functionally, pharmacological caspase-8 inhibition confers clear benefits in OA chondrocytes - including increased viability and proliferation, reduced senescence, and decreased MMP-13 release without inducing apoptosis - whereas non-OA cells show minimal changes only, indicating a pathology-driven effect. Notably, contrary to pharmacological inhibition, knockdown of caspase-8 mRNA by siRNA does not influence functional data. This might be because genetic deletion and catalytic-site inhibition do not address exactly the same biological question. Caspase-8 has not only catalytic functions, but also non-catalytic domain-dependent functions, particularly scaffolding-like roles mediated by its death effector domains (DEDs). This conceptual distinction is also supported by recent work in Nature Metabolism which suggests that genetic deletion and catalytic inhibition do not necessarily capture the same layer of biology [20]. Therefore, in the context of our present question -whether caspase-8 catalytic activity contributes to OA-related phenotypes- selective catalytic inhibition is not necessarily weaker than genetic ablation, and provides a more direct mechanistic readout.

Notably, treatment of chondrocytes with Z-IETD-FMK not only reduced caspase-8 activity but also caspase-1 and − 9 activity, Reduction of caspase-1activity, which is a prototypical inflammatory caspase activated within canonical and non-canonical inflammasome complexes, respectively, can cleave and activate pore-forming gasdermin D (GSDMD) to cause pyroptotic cell death [21]. Caspase-8 can regulate inflammasome activation [22] and that way affect caspase-1 activity. In addition, reduced caspase-9 activity – the key initiator caspase of the intrinsic pathway – after caspase-8 inhibition may actually not be too surprising, given the canonical cross-talk between extrinsic and intrinsic apoptotic pathways, whereby caspase-8 inhibition could reduce BID-mediated mitochondrial outer membrane permeabilization and thereby secondarily decrease caspase-9 activation [23–25].

Our results indicate a functional shift of caspase-8 in OA chondrocytes from a canonical apoptosis initiator to a primarily non-apoptotic mediator of inflammation, senescence, and fibrosis. Mechanistically, this aligns with the concept that articular chondrocytes behave as Type II apoptotic cells [7, 26], in which extrinsic apoptosis signals driven by death receptor-mediated caspase-8 activation are too weak to induce cell death on their own and must be amplified through the intrinsic, mitochondria-dependent apoptosis pathway to execute apoptosis [27, 28]. In such a context, preserving mitochondrial integrity becomes the key decision point for apoptosis, and upstream inhibition of caspase-8 alone is unlikely to fully block apoptosis once mitochondrial damage has occurred. This framework helps reconcile apparently conflicting in vivo findings: in the ACLT post-traumatic OA rabbit model, caspase-8 inhibition with the peptide-based inhibitor Ac-IETD-CHO failed to significantly modify the disease, whereas combined inhibition of caspase-8 and caspase-3 showed chondroprotective effects [9].

In this context, blockade of caspase-8 activity may not effectively prevent apoptosis, however depending on the applied pharmacological inhibitor type. Application of Z-IETD-FMK reduces not only caspase-8 activity but also apoptosis executing caspases-3/7 activity significantly indicating an indirect inhibitor effect as caspases-3/7 act downstream of caspase-8. Moreover, the rabbit ACLT model represents an injury-driven form of OA that differs from spontaneous, age-associated human OA, which is characterized by chronic low-grade inflammation, cellular senescence, and progressive cartilage phenotypic remodeling, including fibrotic changes [1, 29]. Thus, the limited efficacy of caspase-8 inhibition observed in the ACLT model does not necessarily preclude caspase-8 as a therapeutic target in spontaneously occurring human OA. In addition, clinical observations in caspase-8–deficient patients further underscore species differences, as caspase-8 loss is embryonically lethal in mice [30] but compatible with survival and immunodeficiency in humans [31], indicating species-specific compensatory mechanisms that constrain the extrapolation of murine findings to human disease. Together, these observations underscore the importance of the type of caspase inhibitor and to investigating caspase-8 in human-derived, disease-relevant contexts.

In experimental OA models which are driven largely by acute mechanical overload, caspase-8 can be bypassed and downstream executioners as caspases-3/7 can be activated directly by activated caspase-9. This presumably eliminates the receptor-mediated extrinsic pathway of apoptosis initiation, and favors the intrinsic pathway of apoptosis, via release of mitochondrial cytochrome c [32]. By contrast, the non-apoptotic phenotypes we describe - sustained inflammation, senescence, and fibrosis - are more characteristic of spontaneous and age-related, low-grade inflammatory OA, where caspase-8 may play a more prominent pathogenic role.

Our multi-omics integration highlights a persistently active DISC–Caspase-8 axis in OA cartilage. Although caspase-8 is upregulated in specific chondrocyte subpopulations, this does not translate into a coordinated activation of downstream apoptotic caspases. Instead, we observe a tighter association with NF-κB and TGF-β signaling, suggesting that chondrocytes divert death receptor signals toward survival and pro-inflammatory pathways.

This is in line with the broader literature showing that caspase-8 functions not only as a protease but also as a scaffold that organizes signaling complexes, such as the FADDosome, to drive NF-κB activation even when its catalytic activity is dispensable [14]. Our data extend this concept to OA pathology: chondrocytes appear to “repurpose” caspase-8 from an executioner of cell death to a mediator of chronic inflammatory and catabolic signaling, thereby avoiding immediate cell loss at the cost of long-term functional deterioration.

Chondrocytes represent a particularly relevant extension of this context-dependent framework. In cartilage, both caspase-8 activity and biological roles vary across developmental stages and microenvironmental conditions. For example, the number of caspase-8–positive cells increases progressively from resting to hypertrophic zones in the growth plate, indicating roles in chondrocyte maturation rather than apoptosis [33]. Also, in Meckel’s cartilage, a transient structure removed primarily through autophagy rather than apoptosis, activated caspase-8 colocalizes with beclin-1–positive autophagic regions. Classical executioner caspases, including caspase-3, -6, -7, and − 9, are absent in this setting [34]. Moreover, during endochondral ossification, caspase-8 enrichment in ossification zones does not coincide with apoptotic localization [35], supporting non-apoptotic regulatory roles in cartilage remodeling.

Single-cell analysis further reveals that the consequences of caspase-8 activation are highly cell-type specific. In inflammatory chondrocytes (Infc), caspase-8 activity is tightly coupled to senescence and SASP programs, reinforcing an inflammation–senescence feedback loop and sustaining matrix degradation. In homeostatic chondrocytes (HomC), caspase-8 disrupts cellular equilibrium by enhancing inflammatory and fibrotic signaling without inducing full senescence, and this sustained sublethal stress progressively forces healthy, matrix-maintaining cells to abandon their homeostatic state and adopt catabolic, stress-responsive, and ultimately inflammatory or fibrotic phenotypes - the defining hallmark of a phenotypic drift. In fibrocartilage- like subpopulations (preFC and FC), caspase-8 is linked to fibrotic gene expression and metabolic reprogramming, including altered mitochondrial function and a shift away from oxidative phosphorylation. These observations suggest that caspase-8 serves as a shared upstream node that drives distinct but converging pathological trajectories - toward senescence, chronic inflammation, or fibrosis - depending on the baseline metabolic state of the chondrocyte.

Functionally, pharmacological inhibition of caspase-8 with the selective peptide-based inhibitor Z-IETD-FMK partially reverses key OA-associated defects. In OA chondrocytes, caspase-8 inhibition reduces the senescence marker SA-ß-galactosidase, restores proliferative and migration capacity, and lowers the synthesis and release of MMP-13, latter particularly under TNF-α stimulated inflammatory conditions. Notably, TNF-α increased CASP8 mRNA and protein expression but did not detectably enhance caspase-8 enzymatic activity. This apparent discrepancy suggests that TNF-α may prime chondrocytes toward a CASP8-expressing inflammatory state without necessarily inducing measurable caspase-8 catalytic activation under the conditions tested. Since caspase-8 activity is regulated not only by protein abundance but also by complex formation, post-translational regulation, substrate accessibility, and cellular context, increased CASP8 expression alone may not directly translate into detectable enzymatic activity. In addition, feedback-mediated regulatory mechanisms may have limited sustained caspase-8 activation, thereby contributing to the absence of a significant increase in measurable enzymatic activity. Therefore, we consider that the observed effects of Z-IETD-FMK are more appropriately interpreted as modulation of TNF-α-associated inflammatory and chondrocyte phenotypes, rather than as direct evidence that the inhibitor reverses TNF-α-induced caspase-8 activation.

Furthermore, these improvements are achieved without inducing overt apoptosis – neither induction of caspases-3/7 activity nor apoptosis indicating Annexin V/7-AAD staining-, as supported by proteomic enrichment analyses, indicating that the primary effect of caspase-8 inhibition is the suppression of pathogenic non-apoptotic signaling rather than blockade of essential apoptotic programs indicated by partly increased cell viability of OA chondrocytes. Proteomic and functional analysis supports this interpretation: Z-IETD-FMK predominantly modulates pathways related to proteostasis, chromatin regulation, ECM remodeling, fibrosis, inflammation, and senescence, with no induction of classical apoptotic executors such as caspase-3/7 or pro-apoptotic Bcl-2 family members.

An important mechanistic insight from our proteomic data is the enhancement of proteostasis and autophagy pathways upon caspase-8 inhibition. Caspase-8 is known to suppress autophagy through cleavage of core autophagy proteins, thereby shifting the balance from survival to cell death [36, 37]. In OA chondrocytes, chronic low sub-apoptotic caspase-8 activity may consequently impair autophagy, promote accumulation of damaged proteins and mitochondria, and contribute to senescence. Inhibiting caspase-8 appears to relieve this brake, leading to improved protein quality control and mitochondrial homeostasis. This restoration of proteostasis, combined with changes in chromatin regulators, may underlie the broader “resetting” of chondrocyte homeostasis that we observe at the phenotypic level.

Our experimental evidence is strongly supported by human genetic analyses. Across multiple MR and SMR frameworks using large-scale GWAS datasets, genetically predicted higher CASP8 expression is consistently and causally associated with increased knee (K)OA risk. This effect is directionally robust and reproducible across cis-eQTLs, trans-eQTLs, mQTLs, and genetic instruments quantifying circulating caspase-8 protein, indicating that CASP8-related OA susceptibility arises from multi-layered transcriptional and epigenetic regulation, rather than a single regulatory mechanism. Importantly, the causal instruments extend far beyond cartilage and blood, mapping to numerous GTEx tissues including blood cells, skeletal muscle, peripheral nerves, vascular tissues, and others. Such multi-tissue regulatory signatures highlight that caspase-8 influences OA risks through system-wide biological networks, integrating inflammatory, metabolic, and senescence-related pathways - well beyond the cartilage compartment alone. This supports the increasingly recognized view that OA is a multi-organ, systemic disease, rather than merely a local degenerative process [29, 38]. The SMR/HEIDI results further exclude linkage confounding, demonstrating that the associations reflect a true causal effect of CASP8 itself, rather than neighboring genes or shared haplotypes. These findings position caspase-8 as an independent driver - not a passive biomarker - within the OA genetic architecture. Taken together, the convergence of experimental and genetic evidence places caspase-8 alongside established OA risk genes regulating chondrocyte de-/differentiation, inflammation, mitochondrial dysfunction, and senescence. This integrated framework strongly supports the translational relevance of targeting caspase-8 and suggests that modulating this pathway may offer genuine disease-modifying potential at both local and systemic levels.

These insights have several therapeutic implications. First, while catalytic inhibition of caspase-8 with agents such as Z-IETD-FMK already shows favorable effects on chondrocyte function, the non-apoptotic functions of caspase-8 depend at least in part on its scaffolding role within signaling platforms such as the DISC and the FADDosome [12, 14, 39]. This suggests that next-generation therapeutic strategies may need to target protein–protein interactions and domain-specific structural features of caspase-8 (e.g., DED - Death Effector Domain interactions in pro-caspase-8), rather than relying solely on catalytic inhibition [40, 41]. Notably, our proteomic data indicate that caspase-8 catalytic inhibition is accompanied by increased pro-caspase-8 abundance, pointing to a putative feedback response, even though this regulatory loop has not yet been formally demonstrated. Such compensation may limit the sustained efficacy of catalytic inhibitors and highlights the need to simultaneously target the non-catalytic scaffolding functions of caspase-8. Second, our in vitro and in situ genetic findings highlight the critical importance of CASP8 transcriptional regulation, alternative splicing, and epigenetic control, revealing additional therapeutic entry points at the RNA and chromatin levels. Approaches that modulate CASP8 promoter activity, enhancer accessibility, or isoform balance may complement catalytic inhibitors and enable more precise control of caspase-8 function. Promoter and enhancer regulation can provide highly specific transcriptional control of caspase-8 expression [42], while epigenetic mechanisms offer an additional regulatory layer; targeting DNA methylation or chromatin remodeling may suppress CASP8 expression independently of catalytic inhibition [43]. Moreover, maintaining the appropriate balance of caspase-8 isoforms is particularly important, as different splice variants exhibit distinct functional properties [44].

Third, given the dual roles of caspase-8 in apoptosis and immune regulation, systemic inhibition carries a theoretical risk of promoting necroptosis or impairing immune surveillance [45]. This underscores the need for careful inhibitor dosing, targeted or local delivery strategies, and biomarker-guided patient selection to balance therapeutic efficacy with safety.

### Limitations of the study

Our study fills an important gap in our understanding of caspase-8 roles in OA pathophysiology from a molecular and multi-omics perspective, but several key aspects remain unaddressed. Our in vitro experiments and omics analyses primarily focus on primary human OA chondrocytes, which exhibit substantial donor-to-donor variability compared with standardized small animal models. Consequently, the specific molecular interactions within the caspase-8–mediated inflammation–senescence–fibrosis axis identified here, require further exploration in controlled in vivo systems, particularly in spontaneous OA models that more accurately recapitulate chronic, low-grade inflammatory joint degeneration than post-traumatic models such as ACLT/DMM. Moreover, although we demonstrate pathway-level alterations associated with caspase-8 activity, the precise structural determinants that distinguish its scaffolding and catalytic functions in chondrocytes remain undefined. Future studies should dissect these domain-specific mechanisms, delineate the isoform-specific roles of caspase-8, and identify robust biomarkers reflecting caspase-8 pathway activation in patient samples. Additionally, we plan to incorporate more refined and extended genetic strategies, not only siRNA mediated caspase-8 knockdown but such as knockout via CRISP/Cas9 combined together with rescue experiments, in order to better distinguish the catalytic versus non-catalytic domain-dependent functions of caspase-8 and to strengthen causal inference.

## Conclusion

In conclusion, our in vitro findings and human genetic data support a potential role for caspase-8 in inflammatory, senescence-related, and fibrotic remodeling in OA and in driving chondrocytes from a healthy functional state toward a pathological trajectory state, characterized by chronic malfunctions. We propose that aberrant caspase-8 signaling is a key trigger - rather than a passive consequence - of cartilage degeneration. Nonetheless, given the current lack of in vivo evidence, further studies in more physiologically relevant human-derived models, including organoid-based systems, and, where appropriate, in vivo pre-clinical settings, are required to clarify its mechanistic specificity and translational potential as a candidate disease-modifying target.

## Supplementary Information

Supplementary Material 1.

Supplementary Material 2.

Supplementary Material 3.

Supplementary Material 4.

## Data Availability

The datasets used and/or analyzed during the current study are available from the corresponding author on reasonable request and as supplementary tables.
