# Supplementary material for: Multi-omics and experimental evidence in human chondrocytes identify caspase-8 as a non-apoptotic regulator of inflammatory, senescent, and fibrotic signaling in osteoarthritis
Source: Cell Commun Signal. 2026 Jun 6;24:337. doi: 10.1186/s12964-026-02985-y (PMC13242140; doi:10.1186/s12964-026-02985-y)
Supplement: Supplementary file 3 — Supplementary Material 3. [file 12964_2026_2985_MOESM3_ESM.docx]

**Multi-omics and human chondrocyte evidence identify Caspase-8 as a non-apoptotic regulator of inflammatory, senescent, and fibrotic signaling in osteoarthritis**

Jian Mei^1^, Penghui Wei^2^, Nicole Schäfer^1^, Marianne Ehrnsperger^3^, [Brian Johnstone](https://www.researchgate.net/profile/Brian-Johnstone-3?_tp=eyJjb250ZXh0Ijp7ImZpcnN0UGFnZSI6Il9kaXJlY3QiLCJwYWdlIjoicHVibGljYXRpb24iLCJwcmV2aW91c1BhZ2UiOiJwcm9maWxlIn19)^4^, Eva Matalova ^5,6^, Susanne Grässel^1#^

**Supplementary Figure**


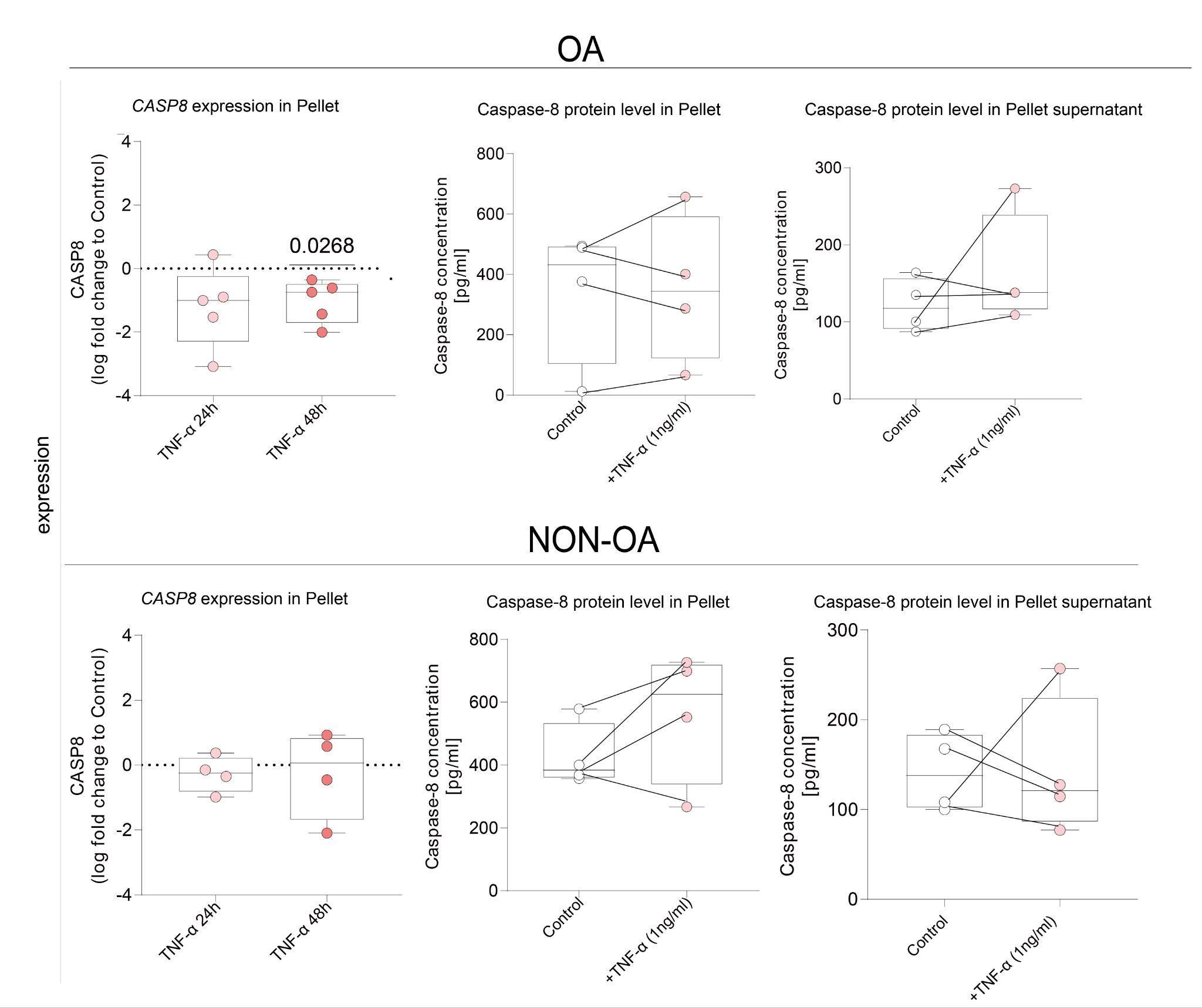


**Suppl. Figure 1.** Effects of TNF-α stimulation on CASP8 gene expression and Caspase-8 protein levels in OA- and non-OA chondrocytes cultured in 3D pellets. CASP8 mRNA expression in cell pellets, intracellular Caspase-8 protein levels, and Caspase-8 levels in culture supernatants were measured following TNF-α (1 ng/mL) stimulation, as indicated. Each dot represents one donor, with paired lines connecting control and TNF-α–treated samples. n =4-5


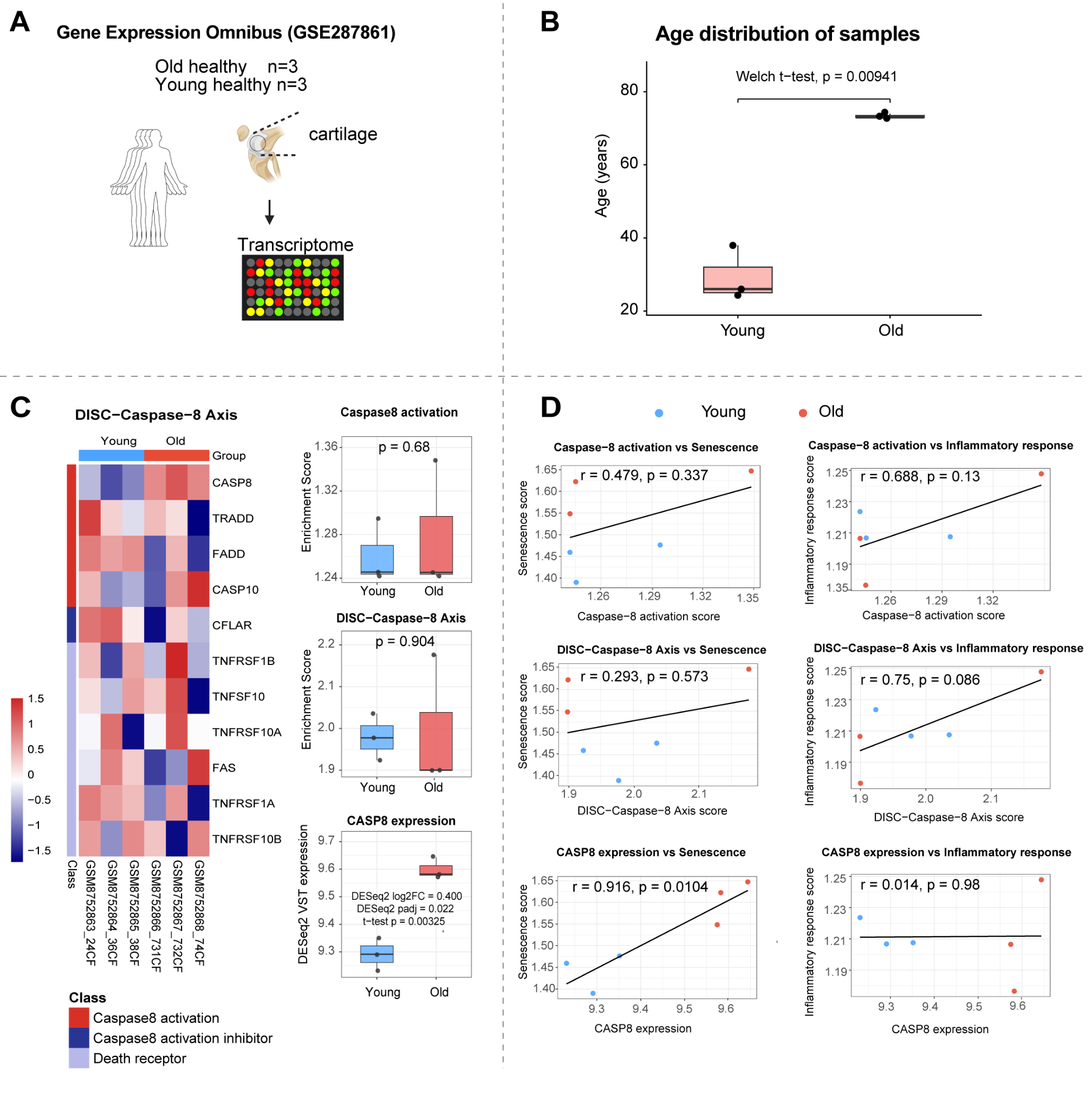


**Suppl. Figure 2.** **Age-based dataset comparing old versus young individuals.** CASP8 expression is associated with senescence-related changes, but not inflammatory response, in an age-based human cartilage transcriptomic dataset. (A) Schematic overview of Gene Expression Omnibus dataset GSE287861, including young and old human cartilage samples (n = 3 per group) used for transcriptomic analysis. (B) Age distribution of the samples.
(C) Heatmap and boxplots showing the expression pattern of the DISC–Caspase-8 axis, the Caspase-8 activation score, the DISC–Caspase-8 Axis score, and CASP8 expression in young and old samples. (D) Correlation plots showing the relationships of the Caspase-8 activation score, DISC–Caspase-8 Axis score, and CASP8 expression with the senescence score and inflammatory response score.


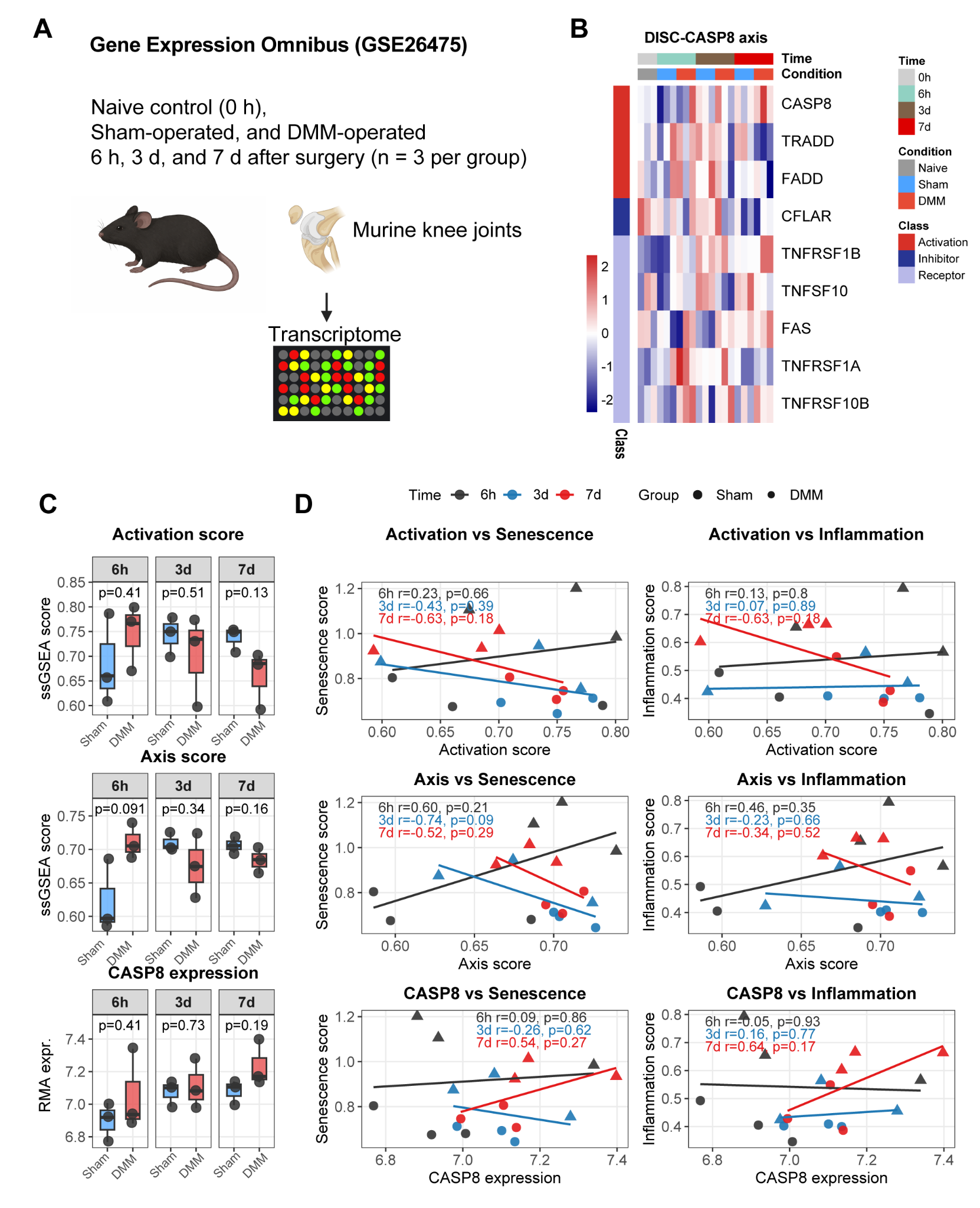


**Suppl. Figure 3. Transcriptomic characterization of the DISC–CASP8 axis in the murine DMM model from GEO dataset GSE26475.** (A) Schematic overview of the Gene Expression Omnibus dataset GSE26475. The dataset includes murine knee joint samples from naive controls (0 h), as well as sham-operated and DMM (destabilization of the medial meniscus) -operated groups collected at 6 hours, 3 days, and 7 days after surgery (n = 3 per group), and was used for transcriptomic analysis. (B) Heatmap showing the expression pattern of DISC–CASP8 axis–related genes across samples. Columns are annotated by time and condition, and genes are grouped by class. Expression values are displayed as z-score–scaled levels. (C) Boxplots showing the activation score, DISC–CASP8 axis score, and CASP8 expression in sham-operated and DMM-operated samples at 6 h, 3 d, and 7 d. P values for the group comparisons are indicated in each panel. (D) Scatter plots showing the correlations of activation score, DISC–CASP8 axis score, and CASP8 expression with senescence score and inflammation score at 6 h, 3 d, and 7 d. Correlation coefficients (r) and P values are shown in each panel.


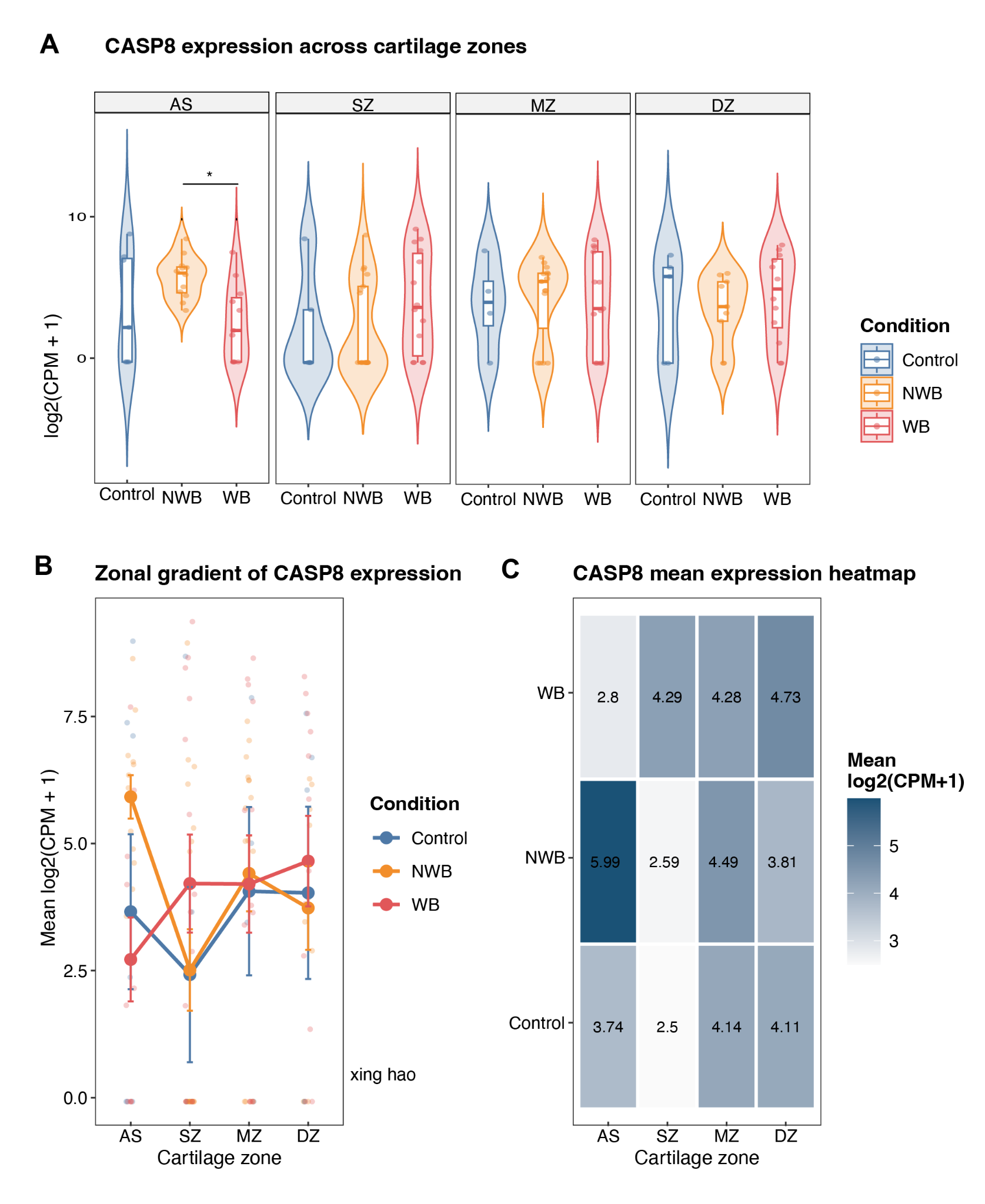
**Suppl. Figure 4. Spatial zonal pattern of CASP8 expression in cartilage.**

CASP8 gene expression was extracted from the processed count matrix, normalized to counts per million (CPM), and transformed as log2 (CPM + 1). (A) Violin plots showing CASP8 expression across the articular surface (AS), superficial zone (SZ), middle zone (MZ), and deep zone (DZ) under control, non-weight-bearing (NWB), and weight-bearing (WB) conditions. Boxplots show the median and interquartile range, and points represent individual spatial samples. Statistical comparisons between conditions within each zone were performed using Kruskal–Wallis tests followed by Dunn’s post hoc tests with Benjamini–Hochberg adjustment; adjusted P < 0.05. (B) Mean zonal expression gradient of CASP8 across cartilage zones. Error bars indicate the standard error of the mean. (C) Heatmap showing mean CASP8 expression for each condition–zone combination, with values shown as mean log2 (CPM + 1). AS, articular surface; SZ, superficial zone; MZ, middle zone; DZ, deep zone; NWB, non-weight-bearing; WB, weight-bearing.


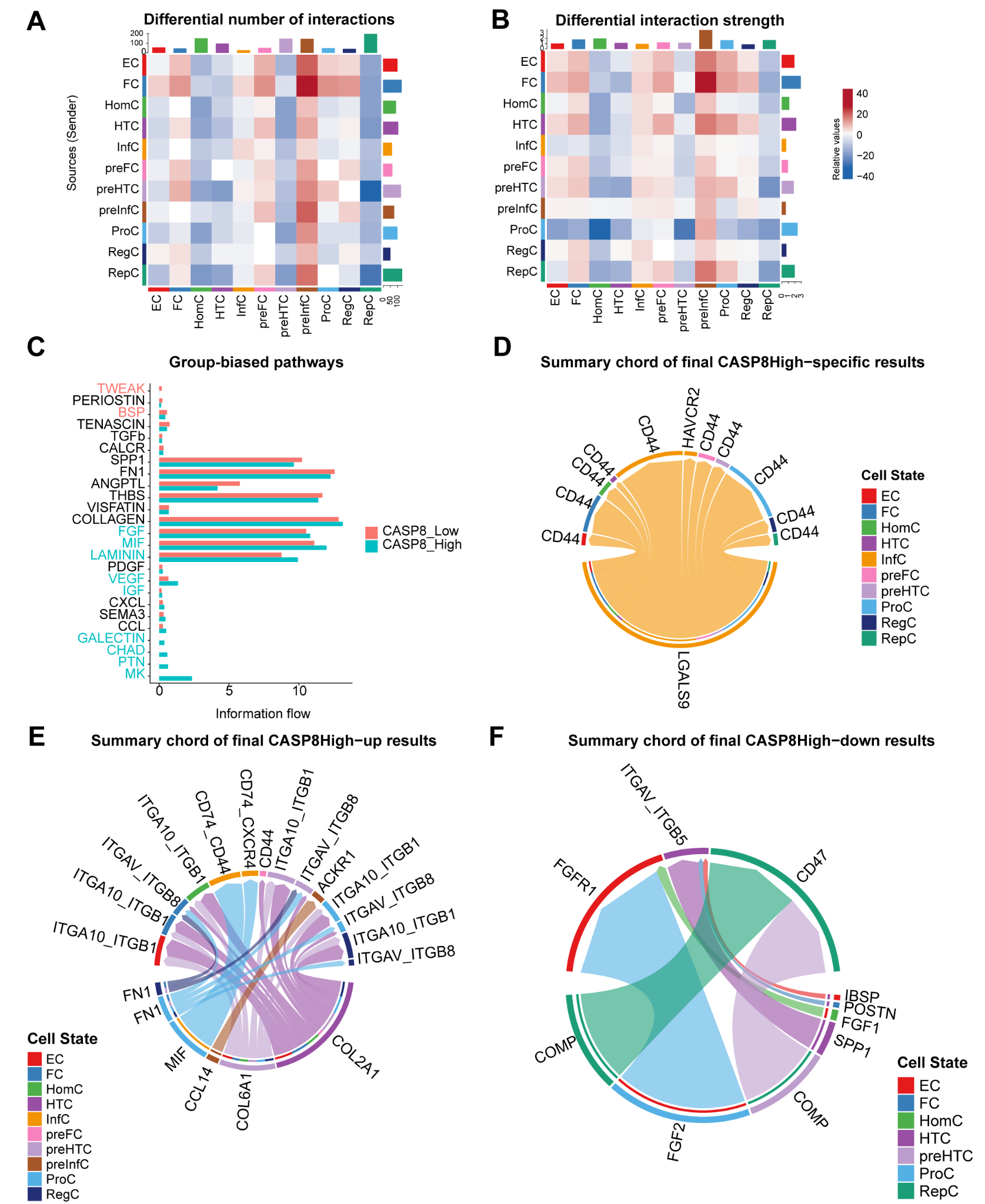


**Suppl. Figure 5. Differential cell–cell communication patterns between CASP8_Low and CASP8_High groups.** (A, B) Heatmaps showing the differential number of interactions (A) and differential interaction strength (B) between sender and receiver cell states in the CASP8_High versus CASP8_Low comparison. Red indicates increased communication in CASP8_High, whereas blue indicates decreased communication. Bar plots summarize the overall outgoing and incoming changes for each cell state. (C) Group-biased signaling pathways ranked by information flow in CASP8_Low and CASP8_High groups. (D–F) Chord diagrams summarizing the final prioritized ligand–receptor interactions after differential and DEG-supported filtering, including CASP8_High-specific interactions (D), CASP8_High-up interactions (E), and CASP8_High-down interactions (F). Colors indicate cell states.


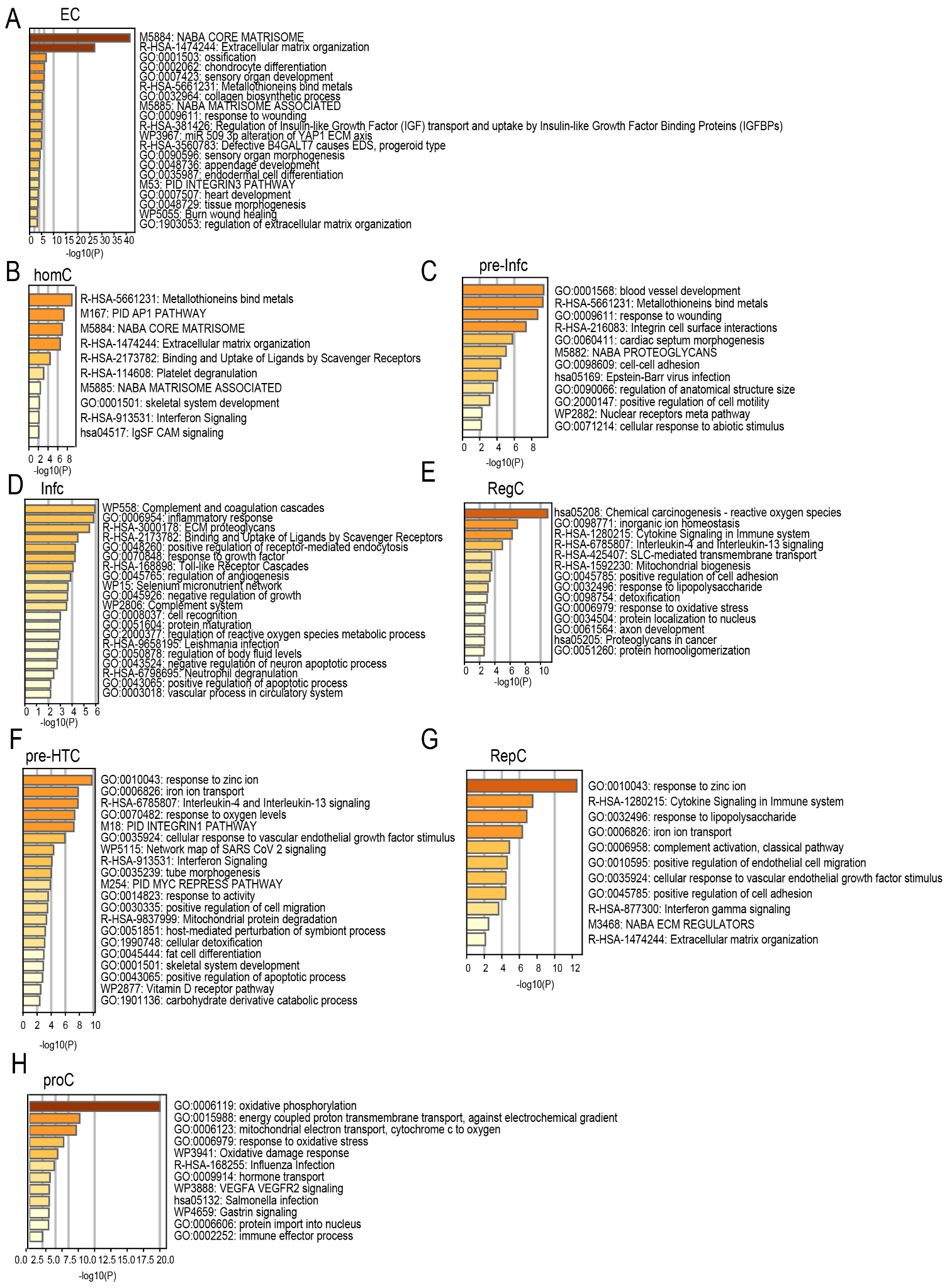
**Suppl. Figure 6. Enrichment analysis of genes altered by in silico CASP8 knockout in single-cell chondrocyte subpopulations.**

(A–H) Bar plots showing the top enriched biological processes and pathways among genes predicted to be altered after in silico CASP8 knockout in EC, homC, pre-InfC, InfC, RegC, pre-HTC, RepC, and proC subpopulations, respectively. Enrichment analysis was performed separately for each subpopulation using Metascape, and significance is shown as −log10(P). Longer bars indicate more significantly enriched terms. The complete gene-level results from the in silico knockout analysis are provided in **Supplementary Table 2**.


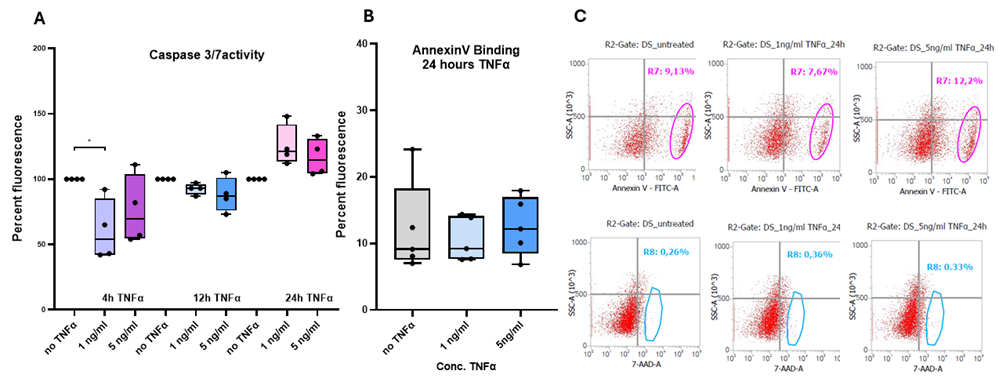


**Suppl. Figure 7. Analysis of apoptotic activity of chondrocytes after treatment with TNFα-** (A) OA chondrocytes were treated with TNFα (1ng/mL or 5ng/mL) for 4h, 12h and 24h. Subsequently, Caspase3/7 activity was measured using the Apo-ONE Homogenous Caspase-3/7 assay. (B) FACS analysis for Annexin V versus 7-AAD staining of OA chondrocytes after 24h TNFα treatment. (C) Representative FACS images for untreated (left) and with 1ng/mL (middle) and 5ng/mL (right) TNFα for 24h treated OA chondrocytes. Upper panel: Annexin V - FITC stained cells area. Lower panel: 7-AAD stained cells area. DS = double – stained; A = area; SSC-A = sideway scatter granularity

Each dot represents a donor. The statistical significance was determined using an Ordinary one-way-ANOVA test followed by Holm-Šídák's multiple comparisons test. *p< 0.05; N=4-5


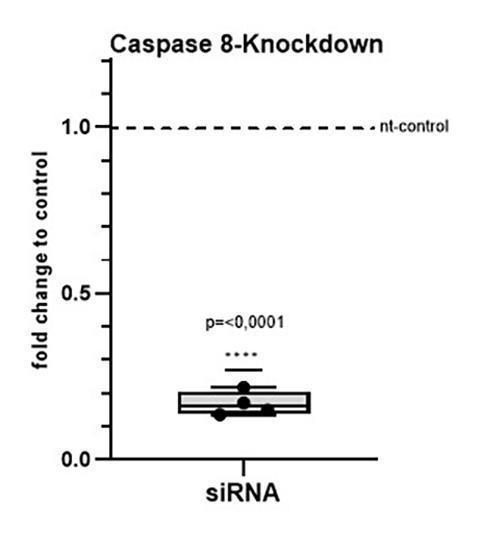


**Suppl. Figure 8. Effect of siRNA mediated knockdown of caspase-8 mRNA on gene expression in OA chondrocytes**. Knockdown efficiency reaches a mean value of 85%, resulting in 15% gene expression compared to the non-target (nt)-control (100%). The nt-control is represented by the dotted line set to 1.0. Each dot represents a donor. Statistical significance was determined using One sample t-test for gene expression analysis; N=4

**
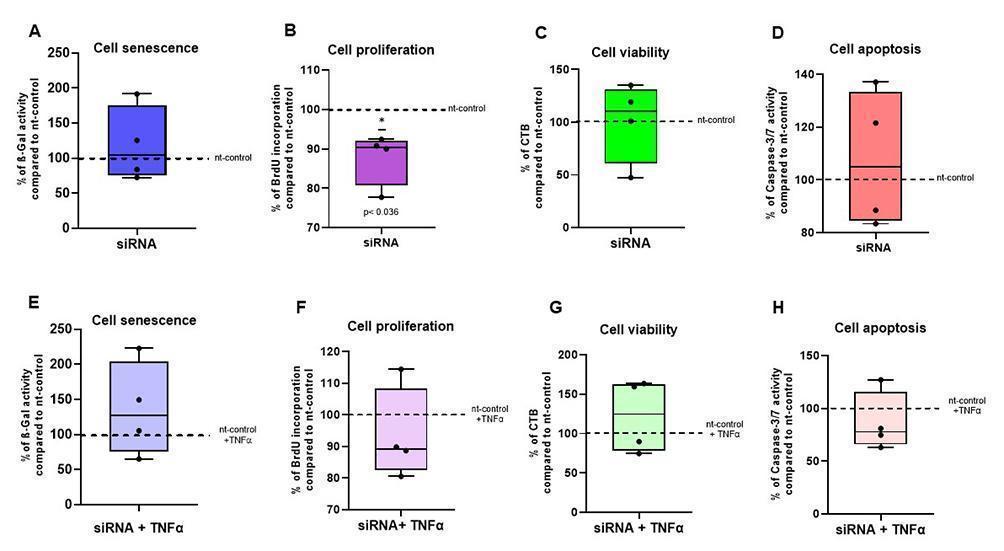
**

**Suppl. Figure 9. Functional assays.** Knockdown of Caspase-8 mRNA via siRNA has no effect on senescence (SA-ß-galactosidase activity), proliferation (BrdU incorporation), viability (CTB assay) and apoptosis (Caspase-3/7 activity) of OA chondrocytes under basal conditions (A-D) and in the presence of TNF-α (1ng/ml) (E-H). Each dot represents a donor. Statistical significance was determined using One-sample t-tests for functional assays. N=4**
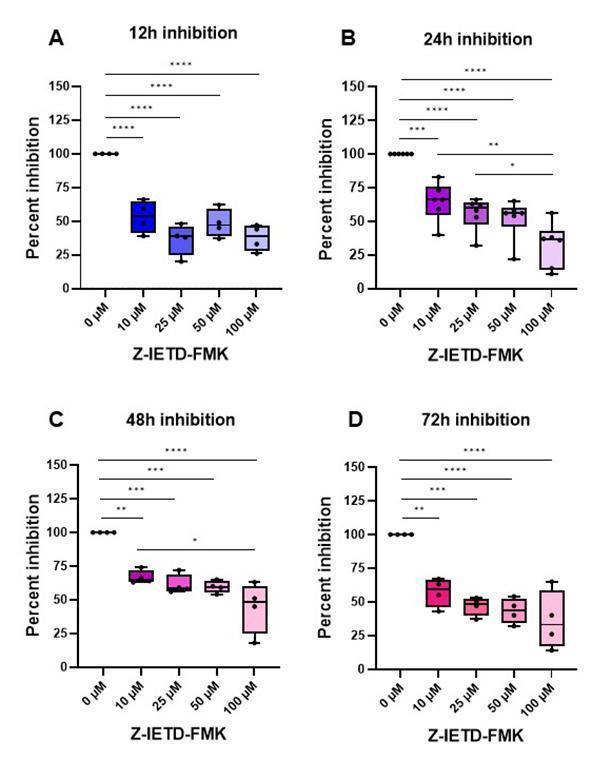
**

**Suppl. Figure 10.** **Titration of Caspase-8 inhibitor Z-IETD-FMK concentration and inhibition time.** OA chondrocytes were treated with caspase-8 inhibitor Z-IETD-FMK in concentrations from 0 uM to 100 µM for 12 (A), 24 (B), 48 (C) and 72 (D) hours. Subsequently, caspase-8 activity was measured with the Caspase-8 Glo® luminescent assay. Each dot represents a donor. The statistical significance was determined using an Ordinary one-way-ANOVA test followed by Holm-Šídák's multiple comparisons test. *p< 0.05; **p< 0.01; ***p<0.001; ****p<0.0001; N=4-6

Caspase-8 activity reduction was achieved with all inhibition time points and inhibition concentrations of Z-IETD-ZMK. However, when using inhibition time points of 24 and 48 hours, the inhibitor concentration of 100µM was most effective, whereas after 12 and 72 hours inhibition time, no statistical differences between the different inhibitor concentrations was detected.


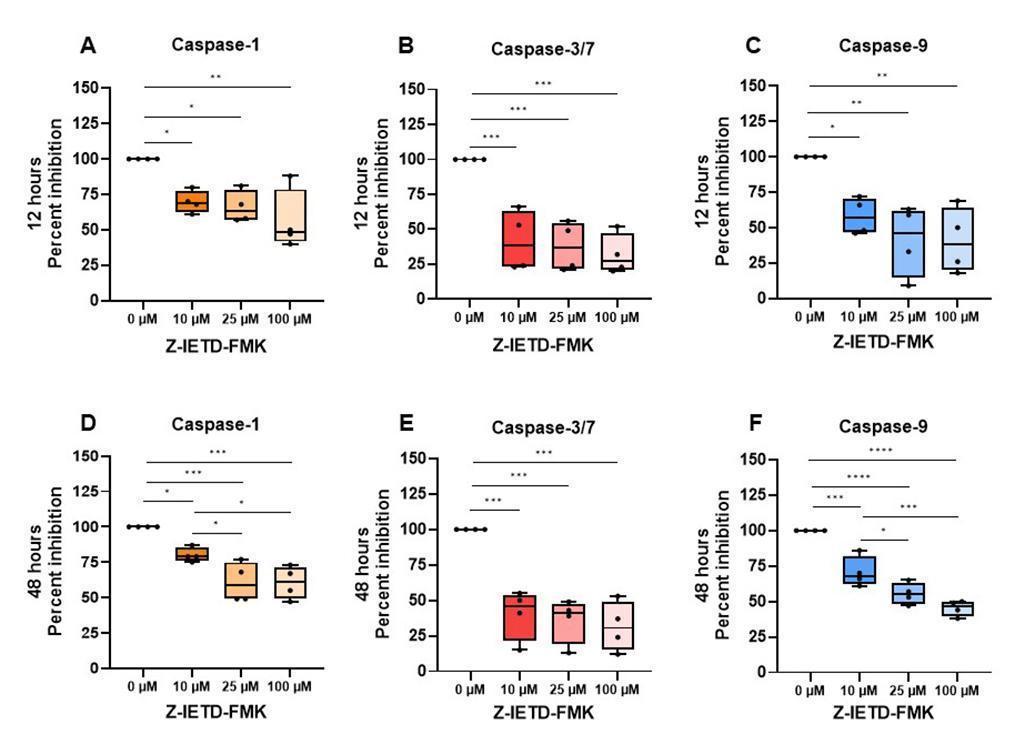


**Suppl. Figure 11.** **Activity of Caspase-1, Caspase-3/7 and Caspase-9 in the presence of Caspase-8 inhibitor.** OA chondrocytes were treated with caspase-8 inhibitor Z-IETD-FMK in concentrations from 0 to 100 µM for 12 (A-C) and 48 (D-F) hours. Subsequently, caspase-1 (A, D), caspase-3/7 (B, E) and caspase-9 (C, F) activities were measured with the corresponding Caspase- Glo® luminescent assays. Each dot represents a donor. The statistical significance was determined using an Ordinary one-way-ANOVA test followed by Holm-Šídák's multiple comparisons test. *p< 0.05; **p< 0.01; ***p<0.001; ****p<0.0001; N=4


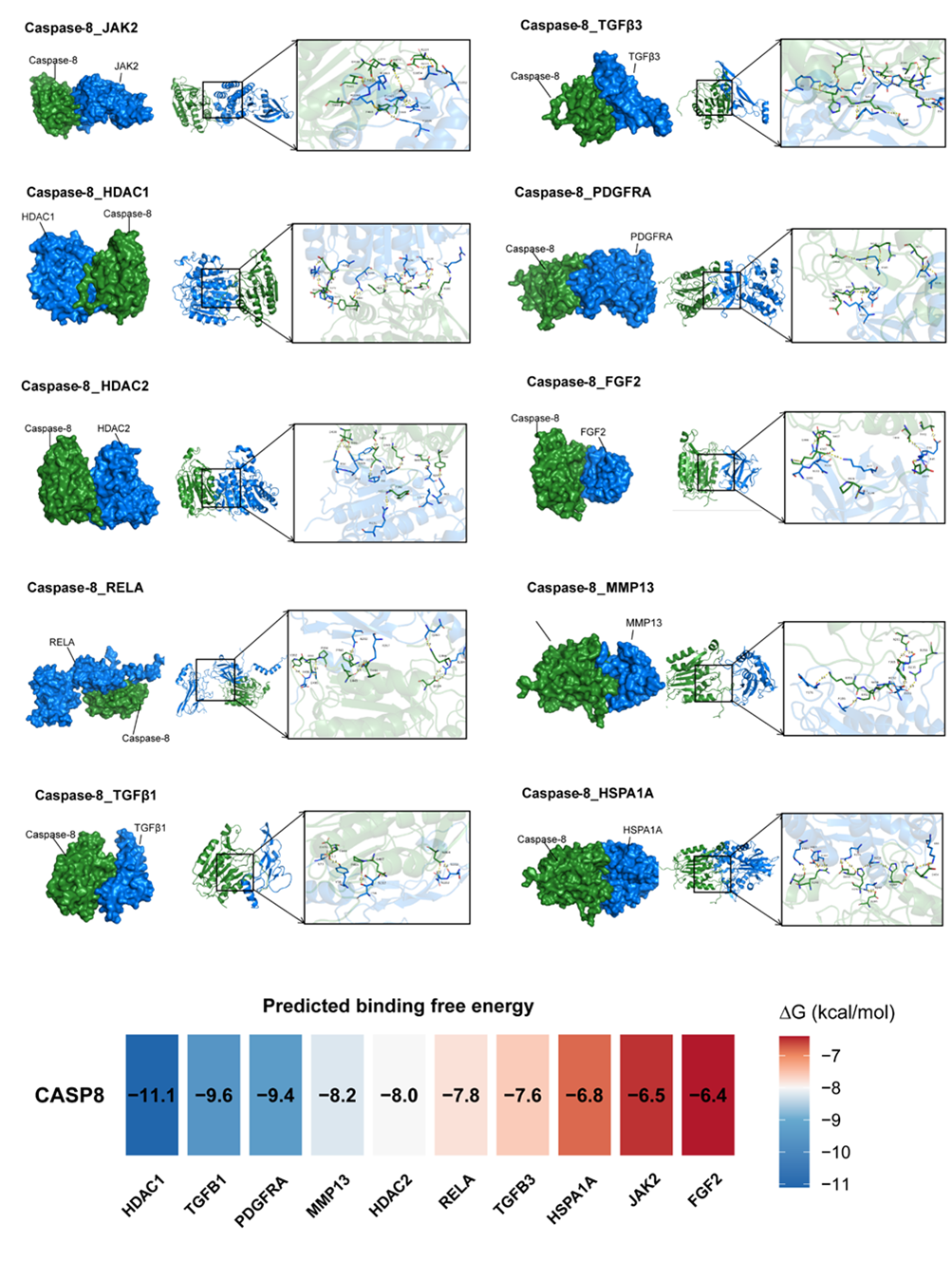


**Suppl. Figure 12. Predicted docking interfaces and binding free energies of Caspase-8 with candidate interacting proteins.** Representative docking models of caspase-8 with JAK2, TGFB3, HDAC1, PDGFRA, HDAC2, FGF2, RELA, MMP13, TGFB1, and HSPA1A are shown, together with enlarged views of the predicted interaction interfaces. Caspase-8 is shown in green and partner proteins in blue. The heatmap summarizes the predicted binding free energy (ΔG, kcal/mol), where more negative values indicate stronger predicted binding.
