# Supplementary material for: Multi-omics and experimental evidence in human chondrocytes identify caspase-8 as a non-apoptotic regulator of inflammatory, senescent, and fibrotic signaling in osteoarthritis
Source: Cell Commun Signal. 2026 Jun 6;24:337. doi: 10.1186/s12964-026-02985-y (PMC13242140; doi:10.1186/s12964-026-02985-y)
Supplement: Supplementary file 4 — Supplementary Material 4. [file 12964_2026_2985_MOESM4_ESM.docx]

**Multi-omics and human chondrocyte evidence identify Caspase-8 as a non-apoptotic regulator of inflammatory, senescent, and fibrotic signaling in osteoarthritis**

Jian Mei^1^, Penghui Wei^2^, Nicole Schäfer^1^, Marianne Ehrnsperger^3^, [Brian Johnstone](https://www.researchgate.net/profile/Brian-Johnstone-3?_tp=eyJjb250ZXh0Ijp7ImZpcnN0UGFnZSI6Il9kaXJlY3QiLCJwYWdlIjoicHVibGljYXRpb24iLCJwcmV2aW91c1BhZ2UiOiJwcm9maWxlIn19)^4^, Eva Matalova ^5,6^, Susanne Grässel^1#^

**Supplementary methods**

***3D Micromass Pellets***

Chondrocytes were trypsinized, counted, and resuspended at 1x10⁵ cells per pellet in complete chondrogenic medium composed of high-glucose DMEM (Life Technologies, Darmstadt) supplemented with ITS+ (Corning, Amsterdam), penicillin/streptomycin, 100 nM dexamethasone (Sigma Aldrich, Taufkirchen, Germany), 10 ng/mL TGF-β3 (Life Technologies, Darmstadt) , 50 µg/mL ascorbate-2-phosphate, 40 µg/mL L-proline, and 1 mM sodium pyruvate (Sigma Aldrich, Taufkirchen, Germany). Cell suspensions (240 µL) were seeded into 96-well conical-bottom plates, centrifuged at 500xg for 5 min, and incubated at 37 °C with 5% CO_2_ for 14 days with medium changes every other day. To induce an inflammatory response, TNF-α (final concentration 1 ng/mL) was added on day 12 (48-hour group) or day 13 (24-hour group), and pellets were harvested on day 14. Unstimulated controls were cultured in parallel without TNF-α.

***RNA and Protein Extraction from 3D Micromass Pellets***

Total RNA and protein were extracted from 3D chondrocyte pellets following established chondrogenic pellet protocols. For RNA isolation, pellets were processed using the MasterPure™ Complete RNA Purification Kit (Epicentre, MC85200) with DNase treatment, followed by ethanol washing and elution in RNase-free water. RNA quantity and purity were assessed using NanoDrop™ spectrophotometry (Thermo Scientific, Darmstadt). For protein extraction, pellets were lysed in RIPA buffer (50 mM Tris-HCl, 150 mM NaCl, 1% NP-40, 0.5% sodium deoxycholate, 0.1% SDS; pH 7.4) supplemented with protease/phosphatase inhibitor cocktails (Roche, 04693132001; 04906837001), clarified by centrifugation (12,000 × g, 15 min, 4 °C), and stored at −80 °C.

### *Analysis of the murine DMM transcriptomic dataset GSE26475*

The murine DMM dataset GSE26475 was analyzed to assess CASP8-related transcriptional patterns in experimental OA. Raw CEL files were downloaded from GEO and processed in R using the oligo package. Data were normalized by robust multi-array average (RMA) normalization at the core transcript cluster level and annotated using mogene10sttranscriptcluster.db. Probe-level values were collapsed to gene-level expression by averaging probes mapped to the same gene symbol.

Differential expression analysis was performed using limma, with contrasts comparing DMM-operated and sham-operated samples at 6 h, 3 days, and 7 days after surgery. Casp8 expression and ssGSEA scores for predefined caspase-8 activation, DISC–caspase-8 axis, senescence, and inflammatory response signatures were calculated from the RMA-normalized gene-level matrix using GSVA. Group differences were assessed using two-sided independent-samples t-tests, and time-stratified Pearson correlation analyses were performed to examine associations between Casp8 expression or pathway scores and senescence or inflammatory response scores.

***Analysis of the age-based human chondrocyte transcriptomic dataset GSE287861***

The public age-based human chondrocyte RNA-seq dataset GSE287861 was analyzed to evaluate CASP8-related transcriptional patterns during chondrocyte aging. This dataset was generated on the GPL16791 Illumina HiSeq 2500 platform and included six female human chondrocyte samples, consisting of three young and three old donors. According to the GEO metadata, the young group had a mean age of approximately 32 years, whereas the old group had a mean age of approximately 73 years. Samples were assigned to young and old groups based on the original sample annotations.

Raw count data and sample metadata were downloaded from GEO and analyzed in R. DESeq2 was used for normalization and differential expression analysis between old and young samples. CPM and variance-stabilizing transformed expression matrices were generated for downstream visualization and pathway-level analyses. CASP8 expression and DISC–caspase-8 axis–related genes were extracted from the processed gene-level matrix, and heatmaps were generated using row-wise Z-score-standardized values.

ssGSEA was performed using predefined caspase-8 activation, DISC–caspase-8 axis, senescence, and inflammatory response gene sets. Senescence and inflammation were represented by the SAUL_SEN_MAYO and HALLMARK_INFLAMMATORY_RESPONSE gene sets, respectively. Group differences were assessed using two-sided independent-samples t-tests, with Wilcoxon rank-sum tests calculated as a non-parametric reference. Pearson correlation analyses were used to examine associations between CASP8 expression or caspase-8-related scores and senescence or inflammatory response scores.

***Ligand–receptor-based cell–cell communication analysis***

To assess whether CASP8 expression was associated with altered intercellular communication, ligand–receptor-based cell–cell communication analysis was performed using CellChat based on the annotated single-cell RNA-seq dataset. Chondrocyte subtypes were used as cell identity labels.

Cells were stratified according to CASP8 expression status. CASP8-positive cells were first defined as cells with detectable CASP8 transcript expression. The median CASP8 expression level among CASP8-positive cells was then used as the cutoff. Cells with CASP8 expression equal to or above this median value were assigned to the CASP8-high group. All remaining cells, including CASP8-positive cells below the median and cells with no detectable CASP8 transcript, were assigned to the CASP8-low group. Thus, the CASP8-low group represented a combined CASP8-low/negative population.

Separate CellChat objects were generated for CASP8-high and CASP8-low groups and subsequently merged for comparative analysis. To focus on extracellular signaling processes relevant to cartilage remodeling, the CellChatDB human database was restricted to the “Secreted Signaling” and “ECM–Receptor” categories. The standard CellChat workflow was used to identify overexpressed genes and ligand–receptor interactions, calculate communication probabilities using the triMean method, and aggregate communication networks at the pathway level.

Overall differences in inferred communication were evaluated by comparing interaction number and communication strength between CASP8-high and CASP8-low groups. Differential interaction networks were visualized using heatmaps and circle plots. Sender and receiver roles of individual chondrocyte subtypes were assessed using signaling role analysis, and pathway-level information flow was compared between groups.

Differential ligand–receptor pairs were extracted from the merged CellChat object and classified as increased or decreased in CASP8-high cells relative to CASP8-low cells. To prioritize more robust interactions, CellChat differential expression-supported ligand–receptor mapping was used to identify ligand–receptor pairs supported by corresponding expression changes. CASP8-high-biased or CASP8-high-specific pathways were further summarized at the source–target and ligand–receptor levels. Final communication results were visualized using bubble plots, chord diagrams, heatmaps, and signaling role plots.

***Spatial transcriptomic analysis of GSE254844***

The publicly available spatial/zonal transcriptomic dataset GSE254844 was analyzed to assess the tissue-level distribution of CASP8 expression across cartilage zones and loading conditions. Metadata and processed count matrices were obtained from GEO and processed in R. Sample annotations were parsed to define cartilage zone, loading condition, and replicate information.

Cartilage regions were classified as articular surface (AS), superficial zone (SZ), middle zone (MZ), and deep zone (DZ). Samples were further grouped according to loading condition as control, non-weight-bearing (NWB), or weight-bearing (WB). Processed counts were normalized to counts per million (CPM), and CASP8 expression was extracted and transformed as log2(CPM + 1) for downstream analysis.

CASP8 expression was compared across cartilage zones and loading conditions using violin/box plots, zonal trend plots, and condition-by-zone heatmaps. Statistical comparisons were performed using Kruskal–Wallis tests followed by Dunn’s post hoc tests with Benjamini–Hochberg correction.

***In silico CASP8 virtual knockout analysis***

To investigate the potential regulatory impact of CASP8 perturbation in OA chondrocytes, in silico virtual knockout analysis was performed using the scTenifoldKnk R package. OA cells were extracted from the Seurat object, and the raw RNA count matrix was used as input. The analysis was conducted separately for each annotated chondrocyte cell type. Cell types with fewer than 10 CASP8-expressing cells were excluded. For each remaining cell type, up to 500 cells were randomly sampled, with CASP8-expressing cells preferentially retained to reduce computational burden. The top 3,000 locally variable genes, calculated from log-transformed counts, were selected for network construction, and CASP8 was included in the input matrix when not already present among these genes. CASP8 virtual knockout was then performed using scTenifoldKnk with 10 constructed sub-networks. Differentially regulated genes after virtual knockout were ranked according to adjusted P values and Z-scores. Genes with adjusted P < 0.05 and |Z| > 1.96 were considered significantly perturbed. Cell-type-specific results were exported and visualized using volcano plots.

Significantly perturbed genes from each analyzed chondrocyte subpopulation were then extracted separately and submitted to Metascape (<https://metascape.org/gp/index.html#/main/step1>) for functional enrichment analysis, with Homo sapiens selected as the reference species. Enriched biological processes and pathways were used to interpret the potential downstream regulatory consequences of CASP8 virtual knockout in a cell-state-specific manner.

***In silico protein–protein docking analysis***

To explore whether proteins altered after Z-IETD-FMK treatment may have potential structural compatibility with caspase-8, in silico protein–protein docking analysis was performed between human caspase-8 and selected proteomically altered candidate proteins. Candidate proteins were selected from the quantitative proteomics results based on their differential regulation after caspase-8 inhibition and their relevance to inflammatory, fibrotic, matrix-remodeling, stress-response, or chromatin-regulatory pathways. The analyzed candidates included TGFB1, TGFB3, FGF2, MMP13, PDGFRA, HDAC1, HDAC2, RELA, HSPA1A, and JAK2. Protein information and reviewed human protein entries were retrieved from UniProtKB/Swiss-Prot. The following UniProt accession numbers were used: CASP8, Q14790; TGFB1, P01137; MMP13, P45452; JAK2, O60674; PDGFRA, P16234; HSPA1A, P0DMV8; HDAC1, Q13547; FGF2, P09038; RELA, Q04206; HDAC2, Q92769; and TGFB3, P10600. UniProtKB provides curated protein sequence and functional annotation resources, including reviewed Swiss-Prot entries.

Protein–protein docking was performed using the HDOCK server (<http://hdock.phys.hust.edu.cn/>), which supports integrated protein–protein docking based on a hybrid strategy combining template-based modeling and template-free docking. HDOCK accepts protein structures or sequences as inputs and ranks predicted complex models using its docking scoring scheme. For each docking pair, caspase-8 was used as one docking partner and each selected candidate protein was used as the second partner. Docking was performed using the default HDOCK settings. For each protein pair, the top-ranked docking models were inspected, and the model with the most favorable docking score was selected for downstream visualization and comparison. Representative docking poses were visualized using PyMOL. The overall docking interfaces and enlarged interaction regions were displayed for each caspase-8–candidate protein complex. Docking scores were summarized across all analyzed protein pairs and visualized as a heatmap. Because protein–protein docking provides computationally predicted structural compatibility rather than experimental evidence of direct binding, the docking results were interpreted as exploratory structural references.
